# Nutrient-cycling microbes in coastal Douglas-fir forests: regional-scale correlation between communities, *in situ* climate, and other factors

**DOI:** 10.3389/fmicb.2015.01097

**Published:** 2015-10-08

**Authors:** Philip-Edouard Shay, Richard S. Winder, J. A. Trofymow

**Affiliations:** ^1^Centre for Forest Biology, Department of Biology, University of Victoria, VictoriaBC, Canada; ^2^Canadian Forest Service, Pacific Forestry Centre, Natural Resources Canada, VictoriaBC, Canada

**Keywords:** DGGE, nitrogen-fixation, temperature, moisture, nitrogen, fungi, carbon, ammonia-oxidizing bacteria

## Abstract

Microbes such as fungi and bacteria play fundamental roles in litter-decay and nutrient-cycling; however, their communities may respond differently than plants to climate change. The structure (diversity, richness, and evenness) and composition of microbial communities in climate transects of mature Douglas-fir stands of coastal British Columbia rainshadow forests was analyzed, in order to assess *in situ* variability due to different temperature and moisture regimes. We compared denaturing gradient gel electrophoresis profiles of fungi (*18S*-FF390/FR1), nitrogen-fixing bacteria (*NifH*-universal) and ammonia-oxidizing bacteria (*AmoA)* polymerase chain reaction amplicons in forest floor and mineral soil samples from three transects located at different latitudes, each transect spanning the Coastal Western Hemlock and Douglas-fir biogeoclimatic zones. Composition of microbial communities in both soil layers was related to degree days above 0°C (2725–3489), while pH (3.8–5.5) best explained shifts in community structure. At this spatial scale, climatic conditions were likely to directly or indirectly select for different microbial species while local site heterogeneity influenced community structure. Significant changes in microbial community composition and structure were related to differences as small as 2.47% and 2.55°C in mean annual moisture and temperature variables, respectively. The climatic variables best describing microbial composition changed from one functional group to the next; in general they did not alter community structure. Spatial distance, especially associated with latitude, was also important in accounting for community variability (4–23%); but to a lesser extent than the combined influence of climate and soil characteristics (14–25%). Results suggest that *in situ* climate can independently account for some patterns of microbial biogeography in coastal Douglas-fir forests. The distribution of up to 43% of nutrient-cycling microorganisms detected in forest soils responded to smaller abiotic gradients than host trees.

## Introduction

Ecological niches are multidimensional; trees therefore have adapted to both above- and below-ground environmental factors, whether abiotic or biotic. There is growing evidence of the close links and specificity between above and below-ground communities (e.g., Wardle et al., 2004; van der Heijden et al., 2008), likely as a result of a complex system where both vegetation and microbes evolve and drive the structure of each other’s communities. However, positive and negative effects are not necessarily correlated above- and below-ground, although both contribute in an additive way to overall fitness (Morrien et al., 2011). Microbial communities provide key ecological functions, are usually well-adapted to a tree species genotype (e.g., Finzi et al., 1998; Ste-Marie and Houle, 2006), but can respond differently than above-ground flora to abiotic stressors (e.g., Nantel and Neumann, 1992; Kranabetter et al., 2009) and potentially to climate change. Previous regional-scale studies of the influence of climate on microbial biogeography in forest soils have been confounded by spatial or plant-related effects (Staddon et al., 1998; Bahram et al., 2012).

Co-adapted soil communities are important because long-term forest growth and resilience depends on below-ground processes such as appropriate organic matter degradation and nutrient cycling (van der Heijden et al., 2008). Microbiota are for the most part responsible for the decay of organic matter and recycling of nutrients from plant inaccessible forms (i.e., N_2_ gas, complex organic polymers) to plant accessible ones (i.e., NH_4_^+^, NO_3_^-^, PO_4_^+^ and organic monomers; Prescott et al., 1993; Elsas et al., 2007; Finlay, 2007; Prosser, 2007). In this study we used polymerase chain reaction (PCR) and denaturing gradient gel electrophoresis (DGGE) as an inexpensive, replicable technique to efficiently screen the principal constituents of nutrient-cycling microbial communities (Nicolaisen and Ramsing, 2002; Vainio et al., 2005; Fierer and Jackson, 2006; Oros-Sichler et al., 2007; Hoshino and Morimoto, 2010) accross climatic gradients within the same forest ecosystem.

We targeted fungi, since they play primary roles in the depolymerisation, decay, and mineralization of nutrients from organic matter, especially in acidic soils (Strickland and Rousk, 2010). Furthermore, ectomycorrhizal fungi help plants uptake nitrogen (N) (Marschner and Dell, 1994), especially in organic forms (Finlay et al., 1992). N-fixing bacteria and ammonia-oxidizing bacteria were also targeted for their respective roles as major contributors of N into forest ecosystems (Vitousek and Howarth, 1991; Glick et al., 1999) and as microbes catalyzing most nitrification in forest soils (Prosser, 2007). Nitrogen is the major limiting nutrient in North American forests; its availability is closely knit to productivity (Vitousek and Howarth, 1991) and its cycling in both plants and microbes is most likely an adaptive trait. Preferential uptake of specific N-forms by tree species or genotypes (Gijsman, 1991; Turner et al., 1993) is likely a result of co-adaption of tree physiology with predominant microbial nutrient-cycling regimes.

This study focused on eastern Vancouver Island, BC, Canada located in the Coastal South Pacific Cordilleran ecoclimatic region (Ecoregions Working Group, 1989), dominated by podzolic soils and the cool temperate wet forests of the WH and DFBEC zones (Meidinger and Pojar, 1991). The east coast of southern Vancouver Island lies within the rainshadow of the Insular Mountains and Olympic Peninsula and is transitional between the WH and DF zones (Supplementary Table S1). The transition zone has been predicted to shift westward with climate change (Hamann and Wang, 2006). Relatively recent disturbances and logging have produced similar-aged mature Douglas-fir stands at sites spanning the range of these BEC zones, providing a unique opportunity to test the biogeography of microbial communities in field sites with similar overstory vegetation composition but different moisture and temperature regimes.

The main objectives of this study were to: (1) determine the biogeography of forest floor and mineral soil microbial communities of interest in similar ecosystems but spanning moisture and temperature gradients along the east coast of Vancouver Island; (2) identify primary *in situ* microclimatic and edaphic and vegetation characteristics contributing to microbial community composition and structure.

## Materials and Methods

### Field Sites and Microclimate Monitoring

**FIGURE 1:**
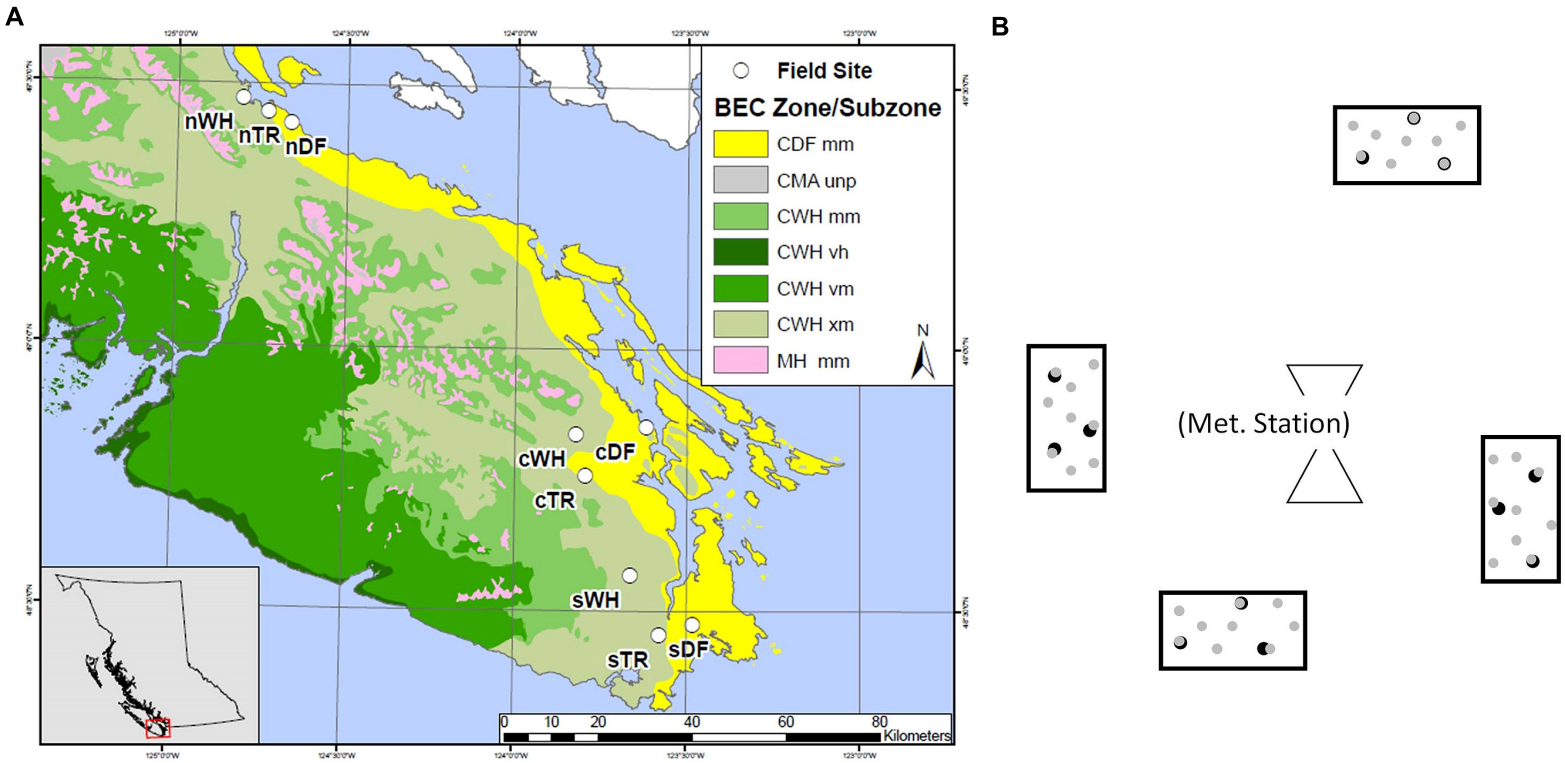
**Layout of southern (s), central (c) and northern (n) transects, each hosting zonal sites in WH, DF and transitional (TR) biogeoclimatic zones.** Map colors depict coastal Douglas-fir (CDF), coastal Western Hemlock (CWH), coastal Mountain-Heather Alpine undifferentiated and parkland (CMA unp) and Mountain Hemlock (MH) Biogeoclimatic (BEC) zones as well as moist maritime (mm), very wet hypermaritime (vh), very wet maritime (vm) and very dry maritime (xm) subzones **(A)**. General plot layout surrounding a meteorological station at a given site, with depiction of the nine forest floor (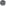) and three mineral soil (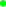) cores in each plot **(B)**.

Forest floor and mineral soil samples were taken from established sites where microclimate has been continuously measured (Saunders et al. unpublished data). Sites were located in zonal WH, DF or Transitional (TR) BEC zones at southern (s), central (c), and northern (n) latitudes between the 48th and 50th parallel (**Table 1**; **Figure 1A**). Each site is within a mesic mature or old Douglas-fir dominated stand. Four rectangular plots (ca. 2.5 m^2^) were set up at random cardinal points and distances (<23 m) from the microclimate station at each site (**Figure 1B**).

**Table 1:** Site location and mean annual microclimatic variables measured from June 15th, 2010 to December 14th, 2012.

Transect	Zone	Latitude	Longitude	Elevation	MS (%)^a^	TA (°C)^b^	TS (°C)^a^	DD^c^	PET^d^
				(m)	(MS_min_, MS_max_)	(TA_min_, TA_max_)	(TS_min_, TS_max_)	(°C days)	(mm)
South	DF	N 48° 28′ 30.4″	W 123° 28′ 58.8″	75	10.8 (0.3, 24.9)	9.3 (-7.7, 32.5)	9.8 (2.3, 17.1)	3245	437
	TR	N 48° 27′ 18″	W 123° 34′ 44.9″	194	11.2 (2.3, 29.6)	8.1 (-11.1, 30)	8.0 (-1.2, 15)	2725	429
	WH	N 48° 34′ 05.2″	W 123° 39′ 46.3″	239	11.4 (2.5, 29.6)	8.3 (-8.9, 28.8)	7.9 (1.7, 18.3)	2964	435
Central	DF	N 48° 51′ 03.8″	W 123° 37′ 13.3″	134	8.5 (1.1, 24.7)	10.5 (-10, 28.5)	9.6 (2.3, 17.4)	3462	461
	TR	N 48° 45′ 32.6″	W 123° 47′ 43.3″	104	7.6 (0, 17.3)	9.7 (-7.7, 30.3)	9.3 (1.3, 17.2)	3344	456
	WH	N 48° 50′ 15.5″	W 123° 49′ 23.5″	236	10.6 (1, 22.8)	8.8 (-8.1, 37)	8.2 (0.7, 15.2)	2891	444
North	DF	N 49° 25′ 30.0″	W 124° 40′ 00.3″	32	10.9 (1, 41.6)	8 (-9.9, 25.4)	9.2 (0.5, 15.7)	3090	431
	TR	N 49° 26′ 47.8″	W 124° 44′ 06.3″	69	11 (0, 49.3)	10.1 (-7.2, 31.3)	9.5 (2.2, 17.8)	3489	452
	WH	N 49° 28′ 22.4″	W 124° 48′ 39.2″	34	11.4 (0.9, 28.1)	8.9 (-10.5, 28.6)	8.8 (1.9, 15.2)	3046	443

*Values in parentheses indicate mean annual minimum and maximum values.*

*^a^Mean annual soil moisture (MS) and temperature (TS) 30 cm below-ground.*

*^b^Mean annual air temperature 30cm above-ground.*

*^c^Degree days with 0°C threshold, calculated from mean monthly temperature.*

*^d^Potential evapotranspiration from May to September, calculated from mean monthly temperature using Thornthwaite method.*

Air and soil temperature as well as soil moisture was logged hourly using Ibuttons (Maxim Integrated, San Jose, CA, USA) and thermocouples and probes connected to a CR10 data logger (Campbell Scientific, Logan, UT, USA), installed either 30 cm above the forest floor or buried 30 cm deep. Three years of moisture and temperature measurements (air and soil) were used to assess microclimates. Vegetation (percent cover of understory species; Supplementary Table S2) and soil edaphic characteristics (see below) of each plot was also recorded. The percent canopy cover of trees (>10 m height) and shrubs (0–10 m height), and ground cover of herbs, seedlings and moss were surveyed at the site level to evaluate similarity of forest stand vegetation and litter input (Supplementary Table S4).

### Soil Sampling and Preparation

To compare the diversity and structure of microbial communities along temperature and moisture gradients, soils were sampled from four replicate plots at each site. Forest floor and mineral soils were sampled once, in September 2011. Composite forest floor (~2 cm-depth) and mineral soil (10 cm-depth) samples were collected using a 5.0-cm-diam. core sampler. Forest floors were mainly mors and composed of a thin layer of litter with underlying F and H layers, at times with intermixed moss, and generally distinct interfaces with mineral soil. To reduce the influence of α-diversity (Martiny et al., 2006; Chowdhury et al., 2011), nine forest floor samples (randomly distributed cores at least 30 cm apart) and three mineral soil samples were collected at each plot (**Figure 1**). Mineral soil cores were collected beneath three of the nine forest floor sample holes, avoiding those exposing large roots and underlying rock. Composite forest floor (177 cm^2^ total surface area) and mineral soil (590 cm^3^ total volume) samples, pooled by plot, were kept in plastic bags on ice in a cooler or refrigerated at 4°C up to 36 h prior to processing.

Each composite sample was separated into coarse and fine particulate fractions by sieving through sterile 8-mm sieves (forest floor organic samples) or 2-mm sieves (mineral soil samples). Coarse mineral soil fractions were further separated into live roots, wood pieces, and gravel. The fine fractions of each sample were homogenized with gloved hands and subsamples (0.25 g) were stored at -20°C for DNA extraction (see below). Dry weight was calculated by air-drying samples for 3 weeks and correcting for moisture content of oven-dried (forest floor samples at 70°C and mineral soils at 105°C) samples (10 g for fine forest floor and 20 g for fine mineral soils).

The pH of air-dried material was measured in aqueous 2 M CaCl_2_ (Kalra and Maynard, 1991) using 10 g subsamples of fine-pulverized forest floor (using a Polymix hammer mill model PX-MFC 90D, Kinematica, Switzerland) or fine mineral soil. Fine-pulverized forest floor or fine-ground mineral soil (using a Siebtechnik GmbH ring grinder, Germany) was dried overnight at 70°C and ~10 mg used for subsequent carbon (C) and N analyses with an elemental combustion system (model ECS 4010 equipped with a dual-column system consisting of a standard reactor packing for C and N followed by a reduction reactor packing, Costech Analytical Technologies Inc, USA). Final %C and %N (mass fraction % in fine soil) of mineral soil samples were corrected for moisture content at 105°C.

### DNA Extraction and PCR-DGGE

DNA was extracted from soil and forest floor samples using the PowerLyser^TM^ PowerSoil^®^ DNA Isolation Kit (MoBio Laboratories Inc., USA) according to manufacturer’s protocol. In order to ensure DNA quantity and quality, extracted DNA was analyzed spectrophotometically using a ND-1000 spectrophotometer (NanoDrop products, USA) and stored at -20°C prior to PCR. DNA concentrations and λ absorbance ratios of 230/280 and 230/260 were compared among sampling sites to ensure unbiased extraction of total DNA.

All primer sets used in this study include a GC-clamp to stabilize the melting behavior of amplified PCR fragments during DGGE (**Table 2**; Sheffield et al., 1987; Kowalchuk et al., 1997). A T-gradient thermocycler (Biometra GmbH, Goettingen, Germany) was used for all PCR steps. Cycling conditions for amplification of the *NifH* gene, used to target bacterial nitrogenase genes, was performed according to modifications by Burgmann et al. (2005) of a protocol by Widmer et al. (1999). Amplification of the *AmoA* gene, for screening of ammonia-oxidizing bacteria (McTavish et al., 1993; Klotz and Norton, 1995; Rotthauwe et al., 1997; Norton et al., 2002; Junier et al., 2009), was performed according to a protocol by Nicolaisen and Ramsing (2002). Amplification of fungal-specific partial fragments of 18S rDNA was performed according to the method of Vainio and Hantula (2000) and Vainio et al. (2005). Pooled DNA from all mineral soil or forest floor samples was used as a PCR template for DGGE ladders, allowing cross-gel comparisons of band migration distances. DGGE was performed using a D-Code^TM^ Universal Mutation Detection system (BioRad Laboratories Ltd., Mississauga, ON, Canada) and conditions outlined in **Table 2**. Gels were electrophoresed for 18 h at 60°C and 80 V, while submerged in 1X TAE buffer (40 mM Tris, 20 mM acetic acid and 1 mM EDTA at pH 8.3), and subsequently stained for 20 min with SYBR^®^ Gold (Invitrogen, Canada). Bands were visualized using a transillumination system (Syngene Chemi-Genius Q Bio-imaging system) and identified using the rolling disk method in GeneTools (Syngene, Frederick, MD, USA) computer software.

**Table 2:** Polymerase chain reaction primer sequences and associated DGGE conditions used in this study.

Target Organisms	Primer	Sequence (5′ → 3′)^a^	Product Size (bp)^b^	(Bis)acrylamide % in DGGE gels	Denaturing gradient^c^	Reference
Fungi	*18S* FF390	CGA TAA CGA ACG AGA CCT	390	7%	30–60%	Vainio and Hantula, 2000
	*18S* FR1^d^	AIC CAT TCA ATC GGT AIT				
β-Proteobacteria Ammonia-oxidizing bacteria	*AmoA* 1F’	GGG GHT TYT ACT GGT GGT	~490	8%	45–70%	Widmer et al., 1999; Burgmann et al., 2005
	*AmoA* 2R^e^	CCC CTC KGS AAA GCC TTC TTC				
Free-living diazotrophs	*NifH*-uni ForA	GCI WTI TAY GGN AAR GGN GG	371	7%	45–70%	Nicolaisen and Ramsing, 2002
	*NifH*-uni ForB^f^	GGI TGY GAY CCN AAV GCN GA				
	*NifH*-uni Rev	GCR TAI ABN GCC ATC ATY TC				

*^a^Degeneracy is indicated by standard conventions: H, not G; Y, C or T; K, G or T; S, C or G; W, A or T; N, A, C, G or T; R, A or G; B, not A.*

*^b^Based on estimates from the literature.*

*^c^Achieved using a gradient-maker (BioRad, Canada) according to manufacturer’s protocol and a 100% denaturing solution composed of 40% deionised formamide and 7 M urea, as defined by Muyzer et al. (1993).*

*^d^GC-clamp: CCC CCG CCG CGC GCG GCG GGC GGG GCG GGG GCA CGG GCC G.*

*^e^GC-clamp: CGC CGC GCG GCG GGC GGG GCG GGG GC.*

*^f^GC-clamp: GC CCG CCG CGC GCG GCG GGC GGG GCG GGG GCA CGG GGG.*

### Data and Statistical Analyses

All statistical analyses were performed using R v3.1.0 (R Development Core Team, 2014).

Concentrations and λ absorption ratios (260/280 and 260/230) of DNA extracted from forest floor and mineral soil samples were modeled in response to transect, zone and their interactions, as well as in response to soil characteristics and vegetation cover, to ensure DNA extraction was not biased by site or environmental factors.

Polymerase chain reaction-denaturing gradient gel electrophoresis fingerprinting cannot detect taxa with proportionally low abundances (<1%), but did not preclude the use of diversity indexes to observe trends of targeted functional groups (Muyzer and Smalla, 1998; Fromin et al., 2002; Fierer and Jackson, 2006; Monroy et al., 2012). Shannon’s diversity (Equation 1), richness (Equation 2), and Pielou’s evenness (Equation 3) of target microbial functional groups were calculated for each plot sample, where n_i_ = peak height of the i^th^ DGGE band, N = sum of peak heights for all bands, and S = total number of bands, and used for analysis of community structure.

(1) $$H'=-\sum \left[\frac{n_{i}}{N}\right]\log\left[\frac{n_{i}}{N}\right]$$

(2) $$d\;=\frac{\left[s-1\right]}{\log[N]}$$

(3) $$J\;=\frac{H^{\prime }}{\log[S]}$$

OTUs found in each sample were also compiled into presence/absence matrices for analysis of community composition.

Constrained and partial-constrained (conditioned) multivariate ordinations were used to differentiate the effects of climatic factors, edaphic characteristics, and spatial distribution on both microbial community composition and structure variables using the ‘vegan’ package (Oksanen et al., 2015). Constrained redundancy analysis (RDA) or constrained correspondence analysis (CCA) was selected for each multivariate model when detrended correspondence analysis of response variables showed a small (<1) or large (>2) range distribution, respectively (Legendre and Birks, 2012). In RDA models, responses were power-transformed when appropriate, to ensure multivariate normality based on skewness (Kankainen et al., 2007). For each model, constraints were selected by backward and forward stepwise elimination of least significant factors based on Akaike Information Criterion (AIC)-like statistics (Godinez-Dominguez and Freire, 2003). The significance of whole models or individual constraints in explaining total Inertia was analyzed by comparing Chi-squared distances (CCA) or Euclidean distances (RDA) of full and reduced models, using permutation tests for RDA or CCA (9999 permutations, pseudo-*F* tests); where constraints are assessed all simultaneously or sequentially. Responses were each scaled to unit variance in all multivariate analyses (see Oksanen et al., 2015 for further details). Microbial functional groups were treated both individually and as a whole, to explore correlated effects between communities. When treating functional groups separately, non-existent community structure indices, resulting from the absence of OTUs in a particular sample, were imputed using the ‘missMDA’ package (Josse et al., 2011) to allow for analysis without the influence of these values.

First, associations between edaphic characteristics or vegetation cover, and climate were assessed, along with those directly between microbial groups. Edaphic characteristics used in multivariate models [fine soil %C and %N and ratios (C/N), pH, and coarse and fine fraction mass and ratio (Co/F); **Figure 3**; Supplementary Tables S3, S7c and S8c] were those significantly different between at least two sites using Tukey HSD univariate pairwise comparisons. In univariate models, edaphic characteristics were transformed, when appropriate, to pass the Shapiro–Wilk normality test. Climate variables included soil moisture variables [mean (MS), minimum (MS_min_), and maximum (MS_max_) annual soil moisture], PET, DD, and temperature variables [mean (TA), minimum (TA_min_), maximum (TA_max_) annual air and soil (TS, TS_min_, TS_max_) temperatures; **Table 1**].

To assess the significant differences in beta-diversity among sites, OTU composition (presence/absence) or community structure (Shannon’s diversity – *H’*, richness – *d*, and Pielou’s evenness – *J*) was analyzed in response to (constrained by) latitude, zone and their interaction. Responses to vegetation cover and edaphic characteristics, climatic factors, or spatial distance were then independently performed to assess best continuous predictors of community composition and structure at various scales, and for comparison with site-level categorical analyses. Spatial distance was parameterized by transforming geospatial coordinates at the plot level into 18 dimensions using PCNM (Borcard et al., 2004). Partial-constrained multivariate ordinations of microbial communities in response to climatic variables (constraints) omitting the effects of edaphic and vegetation heterogeneity (conditions) was also performed, using only factors selected in non-conditioned models, in order to separate correlated environmental effects and assess direct influence of climate on microbial biogeography. Significant climatic and edaphic variables were treated as combined environmental factors in models isolating the effects of spatial distance, as first described by Borcard et al. (1992; Legendre et al., 2005).

## Results

### DNA Quantity and Quality

DNA extractability and quality of both mineral soil and forest floor samples (Supplementary Table S5) did not significantly differ among sites (*P* > 0.05), and was not significantly affected by edaphic characteristics or vegetation cover (*P* > 0.05). Technical replicates of our PCR-DGGE process (Supplementary Figure S1) demonstrated reproducible screening of abundant OTUs of each targeted functional groups.

### Vegetation Cover and Edaphic Characteristics

Canopy/ground cover of trees, shrubs and herbs did not significantly differ with latitude or zone, even when only considering dominant tree species or a single vegetation stratum (Permutation test for RDA *P* > 0.05; data not shown). Nonetheless, canopy cover is less dominated by Douglas-fir and more by Western redcedar and Western hemlock in sTR, nTR and nWH sites (Supplementary Figure S2A). Moss-species composition changed with latitude (Permutation test for RDA under reduced model *P* = 0.006; Supplementary Figure S2B), however, total moss cover was not significantly associated with latitude or zone (ANOVA *P* > 0.05).

**FIGURE 2:**
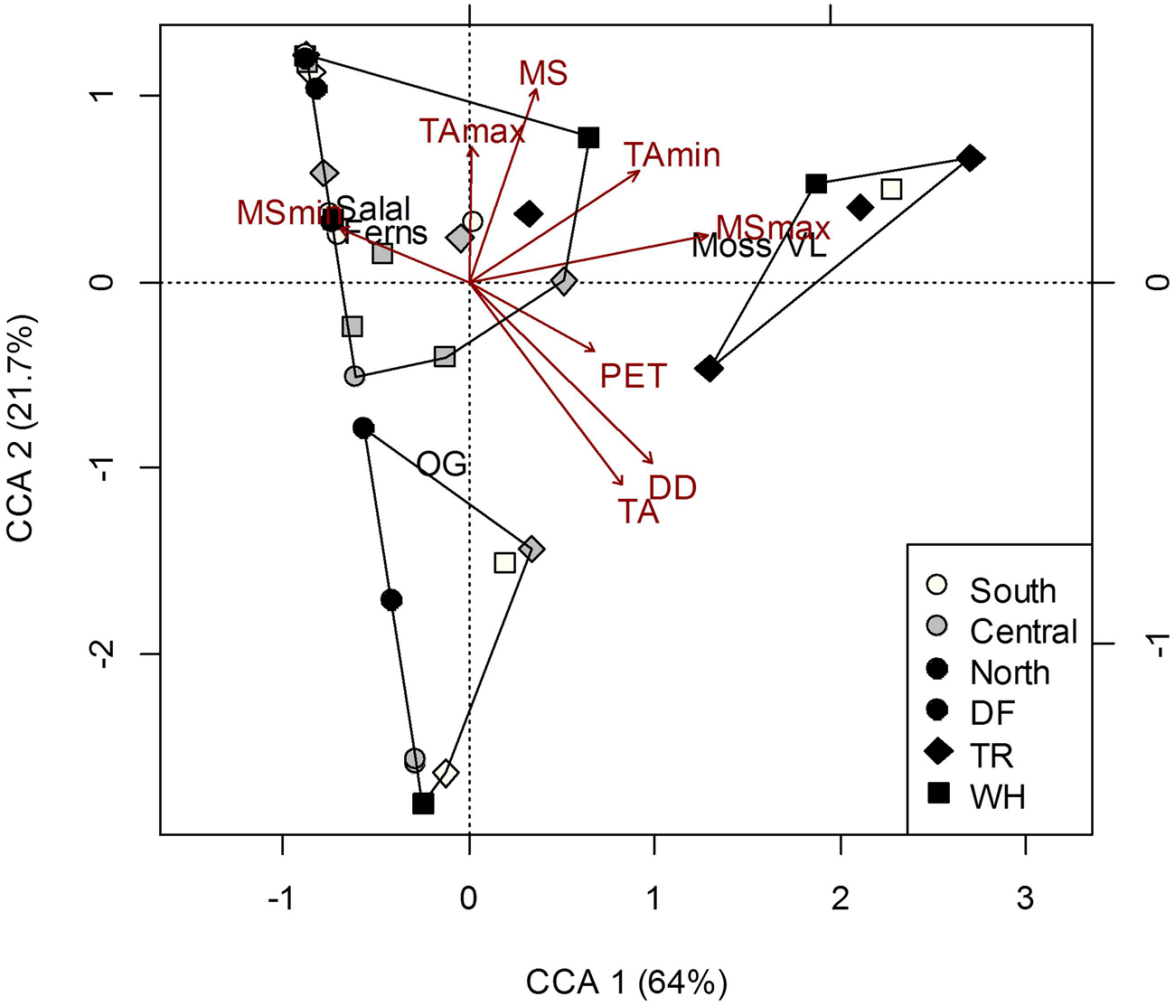
**Constrained correspondence analysis (CCA) of plot vegetation cover [Salal, *Gaultheria shallon*; Ferns, *Polystichum munitum*; Oregon grape (OG), *Mahonia aquifolium*; Vanilla leaf (VL), *Achlys triphylla*; Moss] in response to climate.** Polygons represent k-means clustering using CCA components 1 and 2. All responses were scaled to unit variance. Percent inertia explained by each CCA component is in parenthesis next to axis label. Climate accounted for 34.7% of plot vegetation cover (*P* < 0.05), however, only MS_max_ and PET were significant factors.

Understory vegetation cover at the plot-level was generally quite similar among sites (Supplementary Table S2) and did not show clustering representative of changes in dominant tree cover at the site-level (**Figure 2**). Greater proportion of moss and vanilla leaf cover was associated with most nTR plots, and the relatively high MS_max_ at this site (**Figure 2**; Supplementary Table S6). No relationships were found between forest floor characteristics and vegetation cover (Permutation test for CCAs *P* > 0.05; see Supplementary Table S3 for characteristics of soil layers). To some extent (26.7% of total inertia; Permutation test for CCA *P* = 0.003), more acidic mineral soils were associated with more vanilla leaf and moss, and also less salal, Oregon grape (OG) and fern cover; greater aeration (Co/F ratio) was associated with OG cover, despite pH being negatively correlated with the Co/F ratio (*r* = -0.49; *P* = 0.002).

**FIGURE 3:**
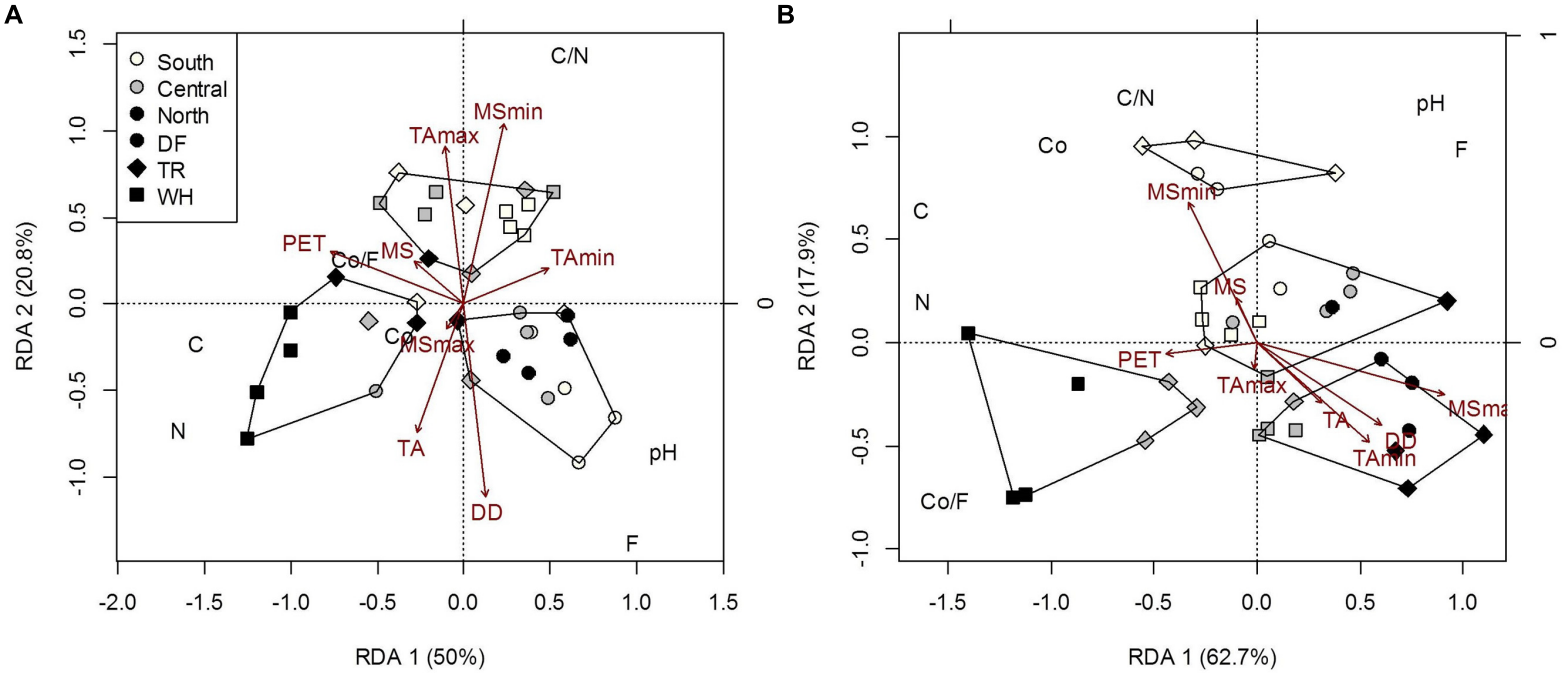
**Constrained RDA of forest floor **(A)** and mineral soil **(B)** edaphic characteristics [Co, coarse fraction content (g); F, fine fraction content (g); Co/F, coarse to fine ratio (g/g); N, nitrogen concentration (%); C, carbon concentration (%); C/N, carbon to nitrogen ratio; pH] of plot samples across latitudes and zones, constrained by climatic variables.** Polygons represent k-means clustering using RDA components 1 and 2. All responses were scaled to unit variance. Percent inertia explained by each RDA component is in parenthesis next to axis label. Climate accounted for 56.7 and 73% of forest floor and mineral soil edaphic characteristics, respectively (*P* < 0.05). MS_max_ and TA were not significant factors in the forest floor model, while MS_min_, PET and DD were not in the mineral soil model.

Edaphic characteristics differed slightly among sites, most prominently C and N (**Figure 3**, Supplementary Table S6). C and N concentrations were highly correlated in all samples (*r* = 0.92 and 0.94 for forest floor and mineral soil, respectively; *P* < 0.001). Climate explained more of the variance in edaphic characteristics in mineral soil (73%) than in forest floor (56.7%) (Supplementary Table S6), indicating more within-site substrate homogeneity in this layer and a potentially stronger relationship to local climate variables. Greater C and N in mineral soil were associated with lower TA_min_ and MS_max_ (**Figure 3**). In both forest floor and mineral soil samples, pH was highest in DF (5.03 and 5.10, respectively), intermediate in TR (4.47 and 4.62, respectively) and lowest in WH sites (4.27 and 4.37, respectively; Supplementary Table S3). Across zones, forest floor pH was lowest in n- and highest in c-transects (Supplementary Table S3).

### Microbial Communities in Forest Floor

**FIGURE 4:**
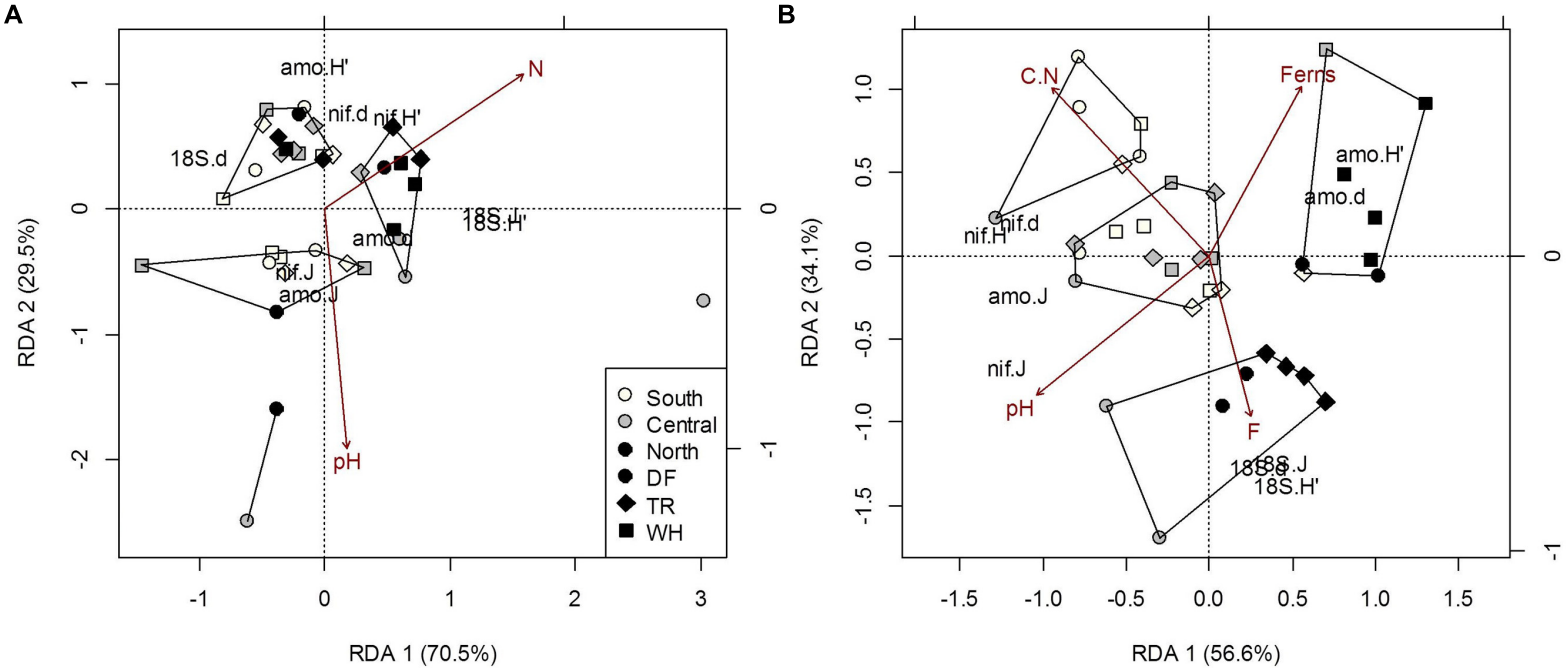
**Constrained RDA of microbial community structure (H’, diversity; d, richness; J, evenness) from forest floor **(A)** and mineral soil **(B)** samples in response to edaphic characteristics and vegetation cover (constraints).** Polygons represent k-means clustering using RDA components 1 and 2. All responses were scaled to unit variance. Percent inertia explained by each CCA component is in parenthesis next to axis label. Microbial functional groups are designated by prefixes 18S, NifH and AmoA for target fungal, nitrogen-fixing and ammonia-oxidizing bacteria communities, respectively. Selected constraints for forest floor samples include pH and nitrogen concentration (N), while those for mineral soil samples include fern cover (Ferns), carbon to nitrogen ratio (C.N), pH and the fine fraction content (F). Constraints accounted for 12.2% and 34.7% of microbial community structure in forest floor and mineral soils respectively (*P* < 0.05).

The direct climatic influences on forest floor microbial community structure along the observed temperature and moisture gradients, as the influences of TA and TA_min_ on fungal and *AmoA* bacteria communities were confounded by the effects of forest floor N (Supplementary Table S7d). Both TA and N had similar effects on fungal community structure, mainly positive correlations with fungal diversity and evenness, and negative correlations with richness (**Figure 4A**; see Supplementary Table S9 for mean OTUs and Shannon’s diversity indices by site). A rise in *AmoA* bacteria richness and evenness, associated with more OTUs in sWH, cDF and nDF samples, can be attributed to generally lower PET and TA_max_ at these sites (Supplementary Table S7b). Fungal community indices increased with increasing TA_max_ and decreasing DD, but these effects were confounded by pH and N (**Figure 4A**; Supplementary Table S7d). Plot differences in the *NifH* bacteria community structure were not associated with latitude or zone, climate or plant cover, but all structural indexes increased with soil C (Supplementary Tables S7a–c) and accounted for 25.9% of trends in *AmoA* community sturcture.

**FIGURE 5:**
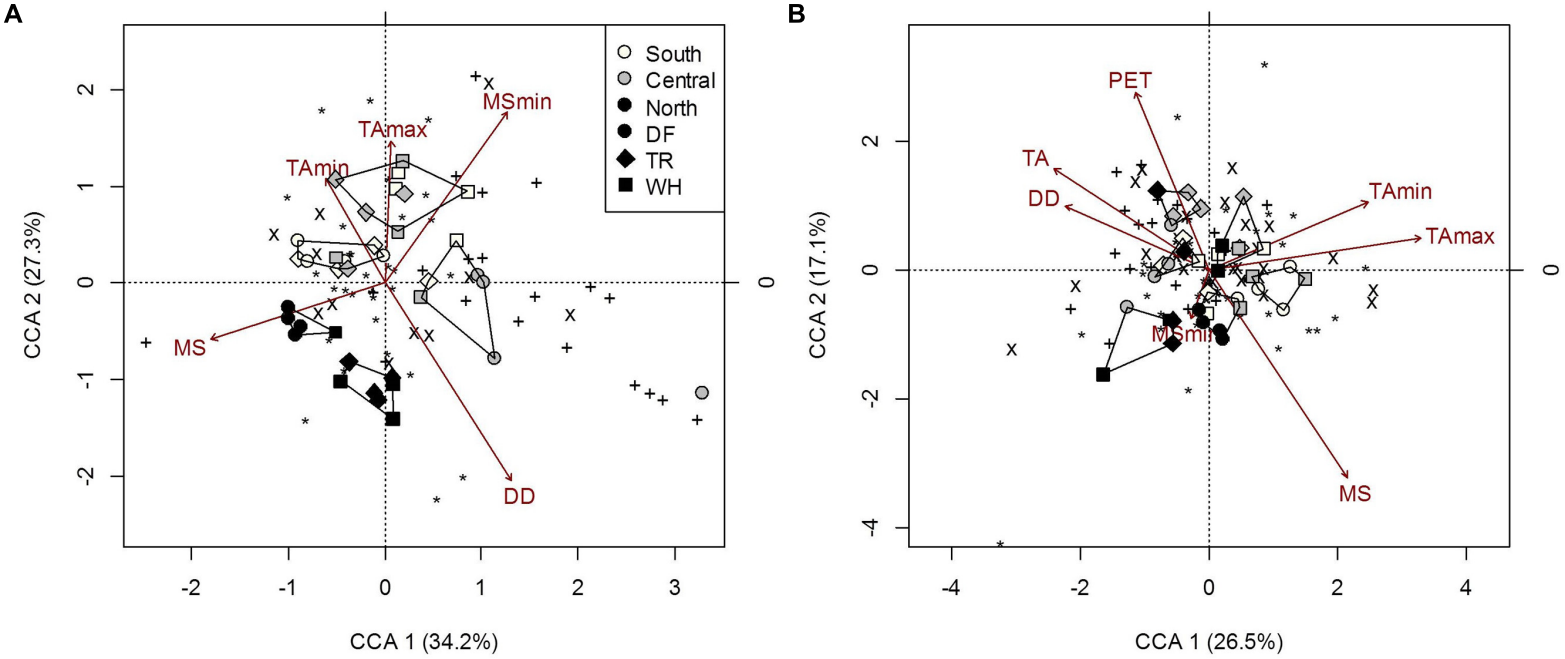
**Constrained correspondence analysis of microbial community composition from forest floor **(A)** and mineral soil **(B)** samples in response to climatic variables (constraints) omitting the effects of edaphic heterogeneity (conditions).** Polygons represent k-means clustering using CCA components 1 and 2. Black symbols each represent a unique OTU (^∗^, *18S-*FF390/FR1; x, *NifH*; +, *AmoA*). All responses were scaled to unit variance. Percent inertia explained by each CCA component is in parenthesis next to axis label. For forest floor, climate variables included MS, MS_min_, DD, TA_min_ and TA_max_, while edaphic characteristics included pH. For mineral soil, climate variables included MS, MS_min_, PET, DD, TA, TA_min_ and TA_max_, while edaphic characteristics included the fine fraction content, [N], [C] and pH. Constraints accounted for 20.4 and 23% of microbial community composition in forest floor and mineral soils respectively, while conditions accounted for 4.9 and 19.4%, respectively (*P* < 0.001).

The overall composition of microbial communities in forest floors was significantly related to MS_min_, DD and temperature extremes, which also separate northern sites from central and southern sites (**Figure 5A**; Supplementary Table S7b). Fungal composition was mainly responsible for microbial community associations with TA_min_ and MS_min_ (**Figure 5A**; Supplementary Table S7b) while both *AmoA* and *NifH* bacteria composition were associated with TA_max_. For the latter, effects were confounded by associations with moss cover and fine fraction content (Supplementary Table S7d). Yet, fungal composition accounted for 91.6% of *NifH* bacteria composition in forest floors (*P* = 0.03). Interestingly, the most significant climatic factor (DD) and edaphic characteristic (pH) describing overall microbial composition did not appear as influential factors when functional groups were examined separately (**Figure 5A**; Supplementary Tables S7b,c); this was possibly an artifact of differences in the number of OTU’s in each group (Supplementary Table S9). Nonetheless, analysis of overall microbial composition clearly isolates two clusters of n-transect samples from other sites (**Figure 5A**).

Although vanilla leaf and moss were the vegetation covers which most differentiated sites, only moss cover was significantly correlated to a microbial community response, namely *NifH* bacteria composition (Supplementary Tables S7b and S8b). Salal cover was significantly linked to *AmoA* bacteria composition when treating functional groups separately (Supplementary Tables S7b and S8b).

### Microbial Communities in Mineral Soil

As with forest floor samples, the confounding effects of edaphic characteristics and vegetation cover made it difficult to directly associate the structure of microbial communities in mineral soil with climatic variables. Community structure differed among latitudes and zones, mainly due to differences in fungi across latitudes and in *NifH* bacteria across latitude and zones. The only significant association directly between microbial communities in mineral soil was fungal community structure accounting for 21.6% of *NifH* community structure (*P* = 0.03). Dryer climates were generally associated with less fungal richness, versus more richness, diversity and evenness of *NifH* bacterial communities (Supplementary Tables S8 and S9). Fungal diversity and evenness differed between southern and northern sites, mostly because of differences between sDF and nDF-nTR sites. Fungal diversity and evenness were 28 and 22% greater, respectively, in sDF than nDF sites (*p* < 0.01 and *p* = 0.011, respectively). These site differences cannot be solely attributed to climate, due to negative correlations with C/N (*r* = -0.49; *P* = 0.002) and positive correlation with fine fraction content (*r* = 0.62; *p* = 0.05) with MS_max_ (**Figure 3**). Northern sites also differed from southern and central sites, as structural indices for *NifH* bacteria decreased with increasing latitude. Richness and evenness of *NifH* bacteria also differed between DF and WH zones (Supplementary Table S8), but the influences of MS and MS_max_ on *NifH* communities were confounded by the effects of C/N and pH, respectively (**Figure 4A**; Supplementary Table S8). The *AmoA* bacteria community structure did not differ significantly among sites; significant associations with DD and MS were confounded by soil pH and fern cover (**Figure 4A**; Supplementary Table S8). Fern cover, which increased with diversity and richness of *AmoA* bacteria, was the only understory vegetation significantly correlated to community structure or composition when modeled responses included all 3 targeted functional groups (**Figures 4B** and **5B**).

The composition of microbial communities in mineral soil was primarily influenced by MS, DD, TA and TA_max_ (**Figure 5A**; Supplementary Table S8). MS was significantly correlated with fungal and *NifH* OTUs (**Figure 5A**; Supplementary Table S8). TA_max_ was primarily associated with changes in fungal composition while DD and TA were associated with changes in *NifH* bacteria composition (**Figure 5B**; Supplementary Table S8). Although TA_min_ correlated with fungal composition in samples, the correlation of TA_min_ with N and C (*r* = 0.44 and -0.41; *P* = 0.007 and 0.014, respectively) is likely responsible for the decreased significance of this climatic factor in the overall microbial composition model when effects of edaphic characteristics are removed (Supplementary Table S8d). The composition of *AmoA* bacterial communities changed with latitude, but this could only be attributed to changes in soil pH (Supplementary Tables S3 and S8). Soil pH also influenced *NifH* bacterial community composition, and accordingly was significantly associated with overall microbial composition (Supplementary Table S8c). Salal cover was only significantly linked to *NifH* bacteria community composition. Other edaphic characteristics influencing overall microbial composition were fine fraction content, C and N, which were associated with changes in both fungal and *NifH* bacterial OTUs (Supplementary Table S8c).

### Microbial Biogeography: Spatial and Environmental

On average, more of the variation in microbial communities was accounted for in mineral soil (47.8%) than in forest floor (33.9%; **Table 3**). Environmental variables (climate, understory cover, and edaphic characteristics) explained between 14 and 25% of variation in microbial community structure and composition. With the exception of mineral soil community structure, that would correspond to 1.3–4.2 times the amount attributable to spatial variables or shared effects (**Table 3**). The highest explanatory power for spatial and shared effects was for mineral soil community structure (22.6 and 16.1%, respectively), where 52.7% of the variance was accounted for by environmental, spatial and shared effects (**Table 3**). On average, shared effects explained 1.8 times more variation in community structure than composition, even though similar variables were used in all models and the number of significant variables was less in community structure models. Transformation of Easting and Northing spatial scales yielded 18 PCNM components, of which only the first five were useful in accounting for microbial biogeography (**Table 3**; see Supplemental Figure S3 for more details). For all microbial community responses except for community structure in forest floor, PCNM 1, which correlates with latitude (*r* = 0.8673, *P* < 0.001), was the only remaining significant spatial scale in models conditioned by their respective environmental variable (*P* < 0.05, 9999 permutations, pseudo-*F* tests). PCNM 2 was the only significant spatial component in environmentally conditioned models of forest floor community structure (*P* < 0.05, 9999 permutations, pseudo-*F* tests), and did not correlate with any of our measured environmental variables.

**Table 3:** Significant environmental variables and spatial principal coordinates of neighbor matrices (PCNM) selected for modeled responses of microbial community structure and composition, and the proportion of Inertia explained by environmental variables alone (Env.), spatial variables alone and a combination of both variables (Both), as well as variance unaccounted for by each model (Unknown).

Responses		Variables		Percent Inertia explained			
Soil layer	Community Index	Environmental factors	Spatial PCNMs	Env.	Both	Spatial	Unknown
Forest floor	Structure	MSmin, TA, TAmin, N	2,4	18.60	11.49	4.46	65.45
	Composition	MSmin, DD, TAmin, TAmax, % salal	1,3,4	14.87	6.67	11.67	66.79
Mineral soil	Structure	MS, MSmax, Fine, DD	1,4,5	13.92	16.15	22.65	47.28
	Composition	MS, MSmin, PET, TA, pH, N, C, Fine	1,3,4	25.53	8.25	9.09	57.13

*All responses were scaled to unit variance.*

## Discussion

Fungal communities were not associated with any changes in vegetation cover, despite numerous documented plant/fungal mutualistic relationships. Very similar host availability among sites is most likely the cause (Tedersoo et al., 2012), however, shifts in fungal communities are not always mirrored by plants (Tedersoo et al., 2014). Root systems were avoided during sampling, on average making up less than 0.4% of sampled mineral soil dry weight, which may have favored the screening of free-living fungal species over mycorrhizal fungi. Overstory needle fall was the primary source of abundant litter at our sites, however, it was not linked to observed site-level trends. Availability of labile organic matter favors mineralization over sequestration (Manzoni et al., 2008; Prescott, 2010) as dissolved organic matter infiltrates into mineral soils. The increase in *AmoA* bacteria diversity and richness in mineral soils with increased fern cover (Supplementary Table S8) concurs with the previously established relationship between sword fern and more nutrient rich sites (Green and Klinka, 1994) and suggests patchy layout of N-sources and sinks at most sites (Ramette and Tiedje, 2007a). The observed correlations between moss cover and the composition of *NifH* bacterial communities in forest floors (Supplementary Table S7) supports other studies showing high diversity and abundance of N-fixing bacteria in moss-dominated ecosystems (Opelt et al., 2007; Winder and Trofymow, unpublished data) and are indicative of nutrient poor sites, likely favoring nitrogen fixation. The lack of significant relationships between fern or moss cover with soil-N or C/N could be due to differences between N-concentrations and N-availability for plants (Schimel and Bennett, 2004); the latter which was not measured. Symbiosis with ericoid mycorrhizae may be responsible for the small but significant association of salal with the composition of *AmoA* and *NifH* in the forest floor and mineral soils, respectively (Marschner and Dell, 1994).

### Microbial Communities in Forest Floor

The composition (i.e., assemblage of OTUs) of microbial communities responsible for decay and N-cycling changed markedly along a latitudinal gradient, but without shifts in community structure (i.e., diversity, richness and evenness indices). At the spatial scales involved, it is possible that climatic conditions in forest floors select for different species while maintaining overall ecosystem functions. Fungal community structure was primarily influenced by edaphic characteristics, which constituted most local heterogeneity. In contrast, fungal community composition was primarily influenced by climatic factors, which changed over larger spatial scales. At least for fungi in the forest floor, climate selected for local population pools, while heterogenous soil matrices determine colonization success for particular microbial species at the site level; confirming findings of Ramette and Tiedje (2007a) concerning the influence of environmental heterogeneity at multiple spatial scales. Spatial distance, whether the result of dispersal limitations or geographic isolation, is an influential factor distinguishing microbial communities (Green et al., 2004; Ramette and Tiedje, 2007b; Monroy et al., 2012) and has likely partially confounded the estimation of other site effects, especially along our latitudinal range. However, our experimental design should mitigate this issue, making distance effects likely attributable to some of the difference in % inertia explained by latitude/zone models and those using climate, edaphic characteristics and vegetation cover (~5% of forest floor microbial composition).

Overall microbial composition was correlated with soil moisture (MS and MS_min_), however, the effects of MS were negated by pH differences. Isolating the direct effects of MS on microbial communities may be difficult since climatic variables such as precipitation, which control soil moisture, also increase the dissociation of acidic fractions from organic litter. This disassociation in turn lowers pH (Brady and Weil, 2008), as seen with the negative correlation of MS and pH (*r* = -0.4239, *P* = 0.01). The observed association of MS_min_ (ranging from 0 to 2.47%) with microbial composition is supported by evidence of varying desiccation tolerances among microbial taxa (Vangestel et al., 1993).

Degree days was an important predictor of overall microbial composition, potentially due to concurrent effects on forest productivity, in agreement with studies of warming-induced feedbacks between above- and below-ground processes (Melillo et al., 2002). Temperature variables were also associated with local *AmoA* bacteria community structure, emphasizing the role of local climate in selecting microbial community assemblages. Climate should therefore be considered as a potential force for aboveground co-adaptation with forest floor microbial communities.

Fungal community structure was affected primarily by N and pH. Soil pH has been shown to be a major predictor of the community structure and composition of bacteria and fungi across biomes (Staddon et al., 1998; Fierer et al., 2009; Strickland and Rousk, 2010; Tedersoo et al., 2014). The observed influence of N on fungal community structure is in accordance with ectomycorrhizal species showing shifts in species dominance along N-gradients (Lilleskov et al., 2001; Toljander et al., 2006; Kranabetter et al., 2009). As more N becomes accessible, a few dominant fungal species tolerant to nutrient stress or specializing in the decay of recalcitrant compounds are likely outcompeted by a richer group of generalists.

### Microbial Communities in Mineral Soil

Soil moisture (MS) was an important predictor of both microbial community structure and composition in mineral soils, especially for N-fixing and ammonia-oxidizing bacteria; however, pH again confounded interpretation of the climatic effect on community structure. Many of the significant differences in community structure were found in the DF zone, where MS negatively correlated with pH (*r* = -0.61; *P* = 0.034). Both factors are likely the result of water regime and its role in cation precipitation and podzolisation (Brady and Weil, 2008). Our ‘mid-scale’ study across two nearby biogeoclimatic zones supports other large-scale studies identifying edaphic characteristics, mainly pH, as the primary control of microbial richness and diversity between ecosystem types (Fierer and Jackson, 2006; Fierer et al., 2009; Lauber et al., 2009; Tedersoo et al., 2014) and findings that soil pH generally affect bacteria more than fungi (Rousk et al., 2010). Although pH differed significantly between WH and DF zones (Supplementary Table S3), its effects were not uniform across functional groups, zone, or soil layer, the latter in contrast with the effects outlined above. Soil pH accounted for fewer changes in microbial composition than local climate, as seen at a global scale in fungal communities (Tedersoo et al., 2014).

Temperature and moisture effects on microbial communities are most likely species- and system-specific (Strickland and Rousk, 2010). Our best climatic descriptors for microbial composition changed from one functional group to the next, but in general did not select for different community structure (Supplementary Table S8). If the community structure of targeted functional groups relates to biological activity, then it is possible that distinct assemblages of microbial populations parallel similar ecosystem functions along climatic gradients within the same forest type. Such selection potentially results from narrower niche widths for some microbes than their host (Nantel and Neumann, 1992; Kranabetter, 2014). Localized co-adaptation of endemic plant populations and microbiota is also a possibility (Kranabetter, 2014), since genotype can have major influences on underground microbiota (Schweitzer et al., 2008). While some of the tree stands at our sites were planted, selected genotypes would have been of locally adapted stock.

Warmer climates tend to have accelerated rates of decay and mineralization (Melillo et al., 2002), which could account for the increased diversity and richness of *AmoA* bacteria with increasing DD at some sites (Supplementary Table S8b). The composition of *NifH* communities was also influenced by DD without effects on general structure (Supplementary Table S8b), further emphasizing the role of temperature in selecting microbial populations along climatic gradients. This process could potentially lead to population endemism. Cosmopolitan gene transfer could also allow for distinct, locally adapted populations to share a common function in response to ecological pressures at a larger scale (Ramette and Tiedje, 2007b). Temperature extremes at the local scale were also shown to play an important role in the selection of microbial populations, namely fungi and *NifH* bacteria (Supplemental Table S8b).

Although we did not measure N-dynamics, mineral soil C/N could be reflective of the N-availability to microbes, since microbial communities shifted from fungi to nitrogen-fixing bacteria with decreasing quality of soil organic matter (**Figure 4B**). Shifts in dominant functional groups and changes in the composition of fungal communities demonstrate significant alterations of microbial niches along N-gradients, further emphasizing the importance of this element for determining trophic cascades (e.g., Knops et al., 2002; Schimel and Bennett, 2004; Kranabetter et al., 2009). Total C has been shown to affect the abundance of forest floor *NifH* bacteria in DF stands with different thinning densities (Levy-Booth and Winder, 2010). We further showed that C significantly correlated with the community structure of forest floor *NifH* bacteria and mineral soil *NifH* bacteria in DF stands along temperature and moisture gradients.

The increases in fungal community structure indices associated with increased fine fraction content in mineral soil may be a result of greater sample size. Although DNA was extracted from subsamples of the same mass, these were taken from homogenized fine fraction content of different sizes (by weight), which may affect the probability of sampling species. Effects of fine fraction content on the community composition of *NifH* bacteria were less clear. Since sample volumes and depth were maintained as constants, fine fraction content was linked to density and therefore could also be related to soil aeration and drainage; however, gravel content and soil compaction complicate this relationship.

### Spatial Implications for Microbial Biogeography

Spatial scales segregating latitude and biogeoclimatic zones are important in determining community structure and composition of nutrient cycling microbes along the rainshadow forests of East Vancouver Island. The spatial component associated with latitude was of particular importance in accounting for microbial variability, in part since spatial distance between s-, c-, and n-transects was greater than distances between zones or plots at each latitude, supporting earlier findings that increasing spatial distance decreases microbial community similarity (Martiny et al., 2006). These effects were particularly pronounced for community structure in mineral soil. Since latitude and zone lose their explanatory power when the effects of environmental variables are removed, PCNM components 3, 4, and 5 likely delineate spatial boundaries correlated to environmental factors significantly affecting microbial communities. Even though these spatial boundaries (i.e., shared spatio-environmental effects) only account for a small fraction of total community variances, they compose a substantial fraction of the explicable community structure (**Table 3**). The generally greater explanatory power of environmental variables therefore does not preclude the importance of spatial and shared spatio-environmental effects on microbial biogeography, especially on species with dispersal limitations (e.g., Tedersoo et al., 2014). The joint influence of climate and spatial distance on nutrient-cycling microbes, even within the same ecosystem type (**Table 3**; Green et al., 2004; Monroy et al., 2012), could infer that ‘naïve’ tree genotypes experiencing climatic shifts or undergoing assisted migration (Winder et al., 2011) have greater exposure to novel soil biota and more potential disconnection from co-adapted communities.

## Conclusion

Climatic and soil factors held the most explanatory power regarding variability of microbial communities in Douglas-fir forest soils. Significant changes in the microbial community composition and structure were related to differences as small as 2.47% and 2.55°C in mean annual moisture and temperature parameters, respectively. The climatic variables best describing microbial composition changed from one functional group to the next, and in general could not be attributed to changes in community structure due to correlations with other environmental characteristics. To a lesser extent, spatial distance, especially associated with latitude, was also important in accounting for community variability. The influence of climate regimes on niche distribution may vary between species and life forms. Special consideration for the interaction of complex above- and below-ground communities is suggested for the development of forest management methods pertaining to climate change adaptation, and warrants further research.

## Author Contributions

PS, RW, and JT conceived and designed the study. PS collected and analyzed the data. PS, RW and JT wrote the manuscript.

## Conflict of Interest Statement

The authors declare that the research was conducted in the absence of any commercial or financial relationships that could be construed as a potential conflict of interest.

## Abbreviations

BEC: biogeoclimatic ecosystem classification
%C: carbon mass fraction % in fine soil
C: central
C/N: mass fraction ratio of carbon to nitrogen in fine soil
Co/F: ratio of coarse to fine fraction mass
DD: degree days above 0°C
DF: coastal Douglas-fir
MS: mean annual soil moisture
MS_min_: mean annual extreme minimum soil moisture
MS_max_: mean annual extreme maximum soil moisture
%N: nitrogen mass fraction % in fine soil
n: nothern
PCNM: principal coordinates of neighbor matrices
PET: potential evapo-transpiration
s: southern
TA: mean annual air temperature
TA_min_: mean annual extreme minimum air temperature
TA_max_: mean annual extreme maximum air temperature
TR: transition between coastal Western Hemlock and coastal Douglas-fir BEC zones
TS: mean annual soil temperature
TS_min_: mean annual extreme minimum soil temperature
TS_max_: mean annual extreme maximum soil temperature
WH: coastal Western Hemlock
