# Supplementary material for: Nutrient-cycling microbes in coastal Douglas-fir forests: regional-scale correlation between communities, in situ climate, and other factors
Source: Front Microbiol. 2015 Oct 8;6:1097. doi: 10.3389/fmicb.2015.01097 (PMC4597117; doi:10.3389/fmicb.2015.01097)
Supplement: Supplementary file 1 [file Data_Sheet_1.DOCX]

***Supplementary Material***

**Nutrient-cycling microbes in Coastal Douglas-fir forests: Regional-scale correlation between communities, *in situ* climate, and other factors.**

**Philip-Edouard Shay^1*^, Richard S. Winder^2^ and J. A. Trofymow^1,2^**

^1^Centre for Forest Biology, Department of Biology, University of Victoria, Victoria, BC, Canada

^2^Canadian Forest Service, Pacific Forestry Centre, Victoria, BC, Canada

*** Correspondence:** P.-E. Shay, University of Victoria, Department of Biology, Centre for Forest Biology, P.O. Box 3020 STN CSC, Victoria, BC, V8W 3N5, Canada

peshay@uvic.ca

1. **Supplementary Figures and Tables**

**1.1 Supplementary Tables**

Table S1: Climatic characteristics for the Coastal Douglas-fir (DF) and Coastal Western Hemlock (WH) biogeoclimatic zones of British Columbia (redrawn from Meidinger and Pojar (1991)). For both zones, the driest and wettest months were July and December, respectively.

| Zone | Range and reference station | Latitude | Longitude | Elevation (m) | Mean annual precip. (mm) | Mean summer precip. (May - Sept) (mm) | Mean percip. of driest month (mm) | Mean precip. of wettest month (mm) | Mean annual snowfall (cm) | No. of months with snow |
| --- | --- | --- | --- | --- | --- | --- | --- | --- | --- | --- |
| DF | Max |  |  | 223 | 1262.6 | 238.3 | 38.6 | 232.9 | 95.2 | 6 |
|  | Min |  |  | 8 | 647.2 | 107.3 | 13.4 | 119.2 | 17.2 | 5 |
|  | Victoria Int'l A | 48°39' | 123°26' | 19 | 872.9 | 141.9 | 18.1 | 157.3 | 49.9 | 6 |
| WH | Max |  |  | 671 | 4386.8 | 1162.0 | 151.0 | 625.4 | 840.8 | 9 |
|  | Min |  |  | 0 | 990.2 | 159.3 | 16.8 | 154.7 | 25.2 | 5 |
|  | Haney UBC RD Admin | 49°16' | 122°34' | 143 | 2140.1 | 467.8 | 65.5 | 331.7 | 81.6 | 7 |
|  |  |  |  |  |  |  |  |  |  |  |
| Zone | Range and reference station | Mean annual temp. (°C) | Mean temp. coldest month (°C) | Extreme min. temp. (C°) | Mean temp. warmest month (°C) | Extreme max. temp. (°C) | No. of frost free days | Frost free period (days) | Degree Days above 5 °C | Degree Days below 0 °C |
| DF | Max | 10.5 | 4.1 | -11.7 | 18.0 | 40.6 | 349 | 304 | 2121 | 43 |
|  | Min | 9.2 | 1.8 | -21.1 | 15.4 | 31.1 | 260 | 155 | 1794 | 9 |
|  | Victoria Int'l A | 9.5 | 3.1 | -15.6 | 16.3 | 36.1 | 305 | 201 | 1863 | 25 |
| WH | Max | 10.5 | 4.7 | -10.6 | 18.7 | 40.6 | 344 | 272 | 2205 | 493 |
|  | Min | 4.5 | -6.6 | -30.6 | 13.1 | 22.8 | 188 | 116 | 1059 | 5 |
|  | Haney UBC RD Admin | 9.2 | 1.4 | -20.0 | 16.8 | 35.0 | 291 | 198 | 1882 | 59 |

Table S2: Percent ground cover of understory vegetation (Salal, *Gaultheria shallon*; Ferns, *Polystichum munitum* and *Pteridium aquilinum*; Oregon grape, *Mahonia aquifolium*; Vanilla leaf, *Achlys triphylla*; Moss) at sampling sites. Each value represents the mean ground cover of 4 sampled plots (±SE).

| Transect | Zone | Salal | Oregon grape | Ferns | Moss | Vanilla leaf |
| --- | --- | --- | --- | --- | --- | --- |
| South | DF | 67.50 ±13.62 | 15.00 ±5 | 11.25 ±11.25 | 6.25 ±6.25 | 0.00 |
|  | TR | 48.75 ±21.05 | 5.00 ±5 | 2.50 ±2.5 | 0.25 ±0.25 | 0.00 |
|  | WH | 51.25 ±25.44 | 17.50 ±15.88 | 0.00 | 28.75 ±22.58 | 0.00 |
| Central | DF | 30.00 ±15.68 | 55.00 ±17.56 | 0.00 | 0.00 | 0.00 |
|  | TR | 42.50 ±14.93 | 26.25 ±8 | 1.25 ±1.25 | 17.50 ±6.29 | 0.00 |
|  | WH | 48.75 ±14.77 | 18.75 ±6.57 | 12.50 ±4.33 | 0.00 | 3.75 ±2.39 |
| North | DF | 41.25 ±18.97 | 22.50 ±13.15 | 8.75 ±4.27 | 0.00 | 0.00 |
|  | TR | 13.75 ±10.48 | 21.25 ±13.29 | 2.50 ±2.5 | 68.75 ±15.99 | 15.00 ±8.66 |
|  | WH | 17.50 ±14.36 | 30.00 ±22.08 | 12.50 ±7.5 | 20.00 ±16.83 | 2.50 ±2.5 |

Table S3: Forest floor and mineral soil characteristics of sampling sites. Carbon and nitrogen concentrations and pH were determined on the fine fraction (<8mm for forest floor and <2mm for mineral soil) of each sample. Mass ratios of coarse to fine fractions (Co/F) and C/N were also calculated for each sample. Each value represents the mean of 4 sampled plots (±SE).

|  |  | Forest floor | | | | | | | | | | | | |  |
| --- | --- | --- | --- | --- | --- | --- | --- | --- | --- | --- | --- | --- | --- | --- | --- |
| Transect | Zone | Coarse fraction (g) | Fine fraction (g) | | | Co/F | | pH | | %N | %C | | C/N | |  |
| South | DF | 16.22 ±7.16 | 232.01 ±18.37 | | | 0.07 ±0.03 | | 4.86 ±0.2 | | 0.49 ±0.04 | 19.84 ±3.07 | | 39.93 ±4.01 | |  |
|  | TR | 36.91 ±14.64 | 108.59 ±21.54 | | | 0.48 ±0.3 | | 4.53 ±0.21 | | 0.57 ±0.1 | 25.79 ±4.55 | | 45.01 ±4.35 | |  |
|  | WH | 19.01 ±5.15 | 102.32 ±13.83 | | | 0.18 ±0.02 | | 4.30 ±0.05 | | 0.38 ±0.02 | 16.98 ±1.28 | | 44.44 ±1.27 | |  |
| Central | DF | 17.03 ±5.75 | 90.93 ±12.86 | | | 0.19 ±0.06 | | 5.25 ±0.1 | | 0.70 ±0.14 | 24.53 ±4.55 | | 35.45 ±1.64 | |  |
|  | TR | 12.46 ±2.78 | 79.40 ±12.37 | | | 0.16 ±0.02 | | 4.64 ±0.21 | | 0.71 ±0.14 | 26.79 ±4.63 | | 38.67 ±2.51 | |  |
|  | WH | 5.28 ±1.23 | 36.19 ±6.85 | | | 0.16 ±0.04 | | 4.44 ±0.19 | | 0.65 ±0.12 | 28.33 ±5.32 | | 43.96 ±0.44 | |  |
| North | DF | 16.37 ±7.49 | 116.83 ±11.19 | | | 0.13 ±0.05 | | 4.98 ±0.03 | | 0.45 ±0.08 | 15.18 ±2.32 | | 34.79 ±2.23 | |  |
|  | TR | 19.61 ±2.7 | 106.31 ±14.02 | | | 0.19 ±0.03 | | 4.26 ±0.09 | | 0.85 ±0.08 | 33.89 ±4 | | 39.81 ±1.41 | |  |
|  | WH | 27.36 ±6.4 | 82.29 ±8.74 | | | 0.37 ±0.12 | | 4.07 ±0.11 | | 1.45 ±0.12 | 43.71 ±0.78 | | 30.65 ±2.11 | |  |
|  |  | Mineral soil | | | | | | | | | | | | | |
| Transect | Zone | Coarse fraction (g) | | Fine fraction (g) | Co/F | | pH | | %N | | | %C | | C/N | |
| South | DF | 527.65 ±132.75 | | 382.47 ±22.25 | 1.34 ±0.32 | | 5.05 ±0.14 | | 0.21 ±0.02 | | | 6.81 ±0.54 | | 33.11 ±1.35 | |
|  | TR | 745.31 ±229.4 | | 478.72 ±99.35 | 1.58 ±0.41 | | 4.75 ±0.2 | | 0.20 ±0.01 | | | 6.85 ±0.62 | | 34.66 ±1 | |
|  | WH | 435.89 ±28.66 | | 279.93 ±23.75 | 1.60 ±0.18 | | 4.40 ±0.11 | | 0.17 ±0 | | | 6.07 ±0.36 | | 35.37 ±1.36 | |
| Central | DF | 302.95 ±39.18 | | 310.55 ±64.4 | 1.10 ±0.23 | | 5.31 ±0.09 | | 0.17 ±0.02 | | | 4.91 ±0.62 | | 28.55 ±1.26 | |
|  | TR | 286.84 ±12.84 | | 97.78 ±27.14 | 3.41 ±0.61 | | 4.54 ±0.13 | | 0.18 ±0.02 | | | 5.72 ±0.75 | | 32.87 ±0.89 | |
|  | WH | 246.44 ±46.32 | | 141.32 ±12.53 | 1.72 ±0.2 | | 4.56 ±0.08 | | 0.15 ±0.01 | | | 4.45 ±0.33 | | 30.24 ±1.62 | |
| North | DF | 228.33 ±88.81 | | 404.94 ±21.36 | 0.57 ±0.21 | | 4.96 ±0.08 | | 0.11 ±0.01 | | | 2.80 ±0.28 | | 26.42 ±1.31 | |
|  | TR | 108.75 ±33 | | 560.30 ±126.14 | 0.20 ±0.04 | | 4.58 ±0.11 | | 0.07 ±0 | | | 1.64 ±0.15 | | 25.19 ±2.45 | |
|  | WH | 685.58 ±197.11 | | 124.94 ±31.57 | 5.70 ±0.97 | | 4.17 ±0.06 | | 0.34 ±0.03 | | | 9.33 ±0.13 | | 27.83 ±1.9 | |

Table S4: Percent canopy / ground cover of vegetation at field sites, and the mean percent presence (P) and cover (MC) of each species across all sites. Species cover was divided into four strata: tree (> 10 m height; A), shrubs (0 - 10 m height; B), herb strata (C) and moss / seedling strata (D).

|  |  |  |  |  |  | South |  |  |  | Central | |  |  | North |  |
| --- | --- | --- | --- | --- | --- | --- | --- | --- | --- | --- | --- | --- | --- | --- | --- |
| Strata | Species | English Name | P | MC | DF | TR | WH |  | DF | TR | WH |  | DF | TR | WH |
| A | *Abies grandis* | grand fir | 11.1 | 0.3 |  |  | 3.0 |  |  |  |  |  |  |  |  |
| A | *Acer macrophyllum* | bigleaf maple | 22.2 | 0.7 |  |  |  |  | 4.0 | 2.0 |  |  |  |  |  |
| A | *Arbutus menziesii* | arbutus | 11.1 | 0.7 |  |  |  |  | 6.0 |  |  |  |  |  |  |
| A | *Cornus nuttallii* | western flowering dogwood | 11.1 | 0.4 |  |  |  |  |  | 4.0 |  |  |  |  |  |
| A | *Pseudotsuga menziesii var. menziesii* | coast Douglas-fir | 100.0 | 43.4 | 60.0 | 25.0 | 50.0 |  | 70.0 | 60.0 | 35.0 |  | 60.0 | 20.0 | 11.0 |
| A | *Thuja plicata* | western redcedar | 55.6 | 16.7 | 5.0 | 50.0 |  |  |  |  | 20.0 |  |  | 30.0 | 45.0 |
| A | *Tsuga heterophylla* | western hemlock | 55.6 | 11.8 |  |  | 5.0 |  | 3.0 |  | 8.0 |  |  | 45.0 | 45.0 |
| B | *Abies grandis* | grand fir | 44.4 | 0.8 | 3.0 |  | 2.0 |  | 1.0 |  | 1.0 |  |  |  |  |
| B | *Acer macrophyllum* | bigleaf maple | 44.4 | 0.8 |  |  | 2.0 |  |  | 1.0 | 1.0 |  | 3.0 |  |  |
| B | *Amelanchier alnifolia* | saskatoon | 22.2 | 0.1 | 0.1 |  |  |  |  | 0.1 |  |  |  |  |  |
| B | *Arbutus menziesii* | arbutus | 11.1 | 0.2 |  |  |  |  | 2.0 |  |  |  |  |  |  |
| B | *Gaultheria shallon* | salal | 100.0 | 55.8 | 52.0 | 60.0 | 85.0 |  | 50.0 | 60.0 | 75.0 |  | 75.0 | 20.0 | 25.0 |
| B | *Holodiscus discolor* | oceanspray | 33.3 | 0.9 | 6.0 |  |  |  |  |  | 1.0 |  | 1.0 |  |  |
| B | *Lonicera hispidula* | hairy honeysuckle | 33.3 | 0.2 |  |  |  |  | 0.1 | 1.0 | 1.0 |  |  |  |  |
| B | *Mahonia nervosa* | dull Oregon-grape | 100.0 | 23.1 | 45.0 | 15.0 | 15.0 |  | 50.0 | 20.0 | 15.0 |  | 15.0 | 8.0 | 25.0 |
| B | *Pinus monticola* | western white pine | 11.1 | 0.1 |  |  | 0.1 |  |  |  |  |  |  |  |  |
| B | *Pseudotsuga menziesii var. menziesii* | coast Douglas-fir | 44.4 | 1.6 | 3.0 |  |  |  | 1.0 | 5.0 |  |  |  |  | 5.0 |
| B | *Rhamnus purshiana* | cascara | 33.3 | 0.1 |  |  |  |  |  |  |  |  | 0.5 | 0.1 | 0.5 |
| B | *Rosa gymnocarpa* | baldhip rose | 66.7 | 0.7 | 2.0 |  | 0.1 |  | 2.0 | 1.0 | 1.0 |  | 0.5 |  |  |
| B | *Rubus spectabilis* | salmonberry | 11.1 | 0.1 |  |  |  |  |  |  |  |  | 1.0 |  |  |
| B | *Symphoricarpos albus* | common snowberry | 22.2 | 0.9 | 4.0 |  |  |  |  | 4.0 |  |  |  |  |  |
| B | *Thuja plicata* | western redcedar | 66.7 | 3.3 |  | 5.0 | 2.0 |  |  | 1.5 |  |  | 10.0 | 3.0 | 8.0 |
| B | *Tsuga heterophylla* | western hemlock | 66.7 | 3.8 |  | 4.0 | 5.0 |  |  | 4.0 | 8.0 |  | 6.0 | 7.0 |  |
| B | *Vaccinium parvifolium* | red huckleberry | 100.0 | 3.6 | 4.0 | 2.0 | 6.0 |  | 3.0 | 2.0 | 2.0 |  | 3.0 | 8.0 | 2.0 |
| C | *Achlys triphylla* | vanilla-leaf | 66.7 | 1.2 |  |  |  |  | 0.1 | 2.0 | 2.0 |  | 1.0 | 5.0 | 0.5 |
| C | *Adenocaulon bicolor* | pathfinder | 22.2 | 0.1 |  |  |  |  |  | 0.1 |  |  | 0.1 |  |  |
| C | *Blechnum spicant* | deer fern | 11.1 | 0.1 |  |  |  |  |  |  |  |  | 1.0 |  |  |
| C | *Carex sp.* | sedge | 11.1 | 0.1 |  |  |  |  |  |  | 0.1 |  |  |  |  |
| C | *Chimaphila umbellata* | prince's pine | 11.1 | 0.2 |  |  | 2.0 |  |  |  |  |  |  |  |  |
| C | *Corallorhiza maculata* |  | 11.1 | 0.1 | 0.1 |  |  |  |  |  |  |  |  |  |  |
| C | *Festuca sp.* | fescue | 11.1 | 0.1 |  |  | 0.1 |  |  |  |  |  |  |  |  |
| C | *Galium aparine* | cleavers | 22.2 | 0.1 |  |  |  |  |  | 0.1 | 0.1 |  |  |  |  |
| C | *Galium triflorum* | sweet-scented bedstraw | 11.1 | 0.1 |  |  |  |  |  |  |  |  |  |  | 0.1 |
| C | *GRASS1* |  | 22.2 | 0.1 |  |  |  |  |  | 0.1 | 0.1 |  |  |  |  |
| C | *LACTMUR* |  | 11.1 | 0.1 |  |  |  |  |  | 0.1 |  |  |  |  |  |
| C | *Linnaea borealis* | twinflower | 11.1 | 0.9 |  |  | 8.0 |  |  |  |  |  |  |  |  |
| C | *Melica subulata* | Alaska oniongrass | 44.4 | 0.1 |  | 0.1 | 1.0 |  |  | 0.1 |  |  |  | 0.1 |  |
| C | *Polygonum minimum* | leafy dwarf knotweed | 11.1 | 0.6 |  |  |  |  |  |  | 5.0 |  |  |  |  |
| C | *Polystichum munitum* | sword fern | 77.8 | 3.4 | 1.0 | 12.0 | 5.0 |  | 1.0 | 5.0 |  |  | 3.0 |  | 4.0 |
| C | *Pteridium aquilinum* | bracken fern | 100.0 | 3.2 | 0.1 | 1.0 | 1.0 |  | 0.1 | 10.0 | 5.0 |  | 5.0 | 6.0 | 1.0 |
| C | *Pyrola asarifolia* | pink wintergreen | 11.1 | 0.1 |  | 0.1 |  |  |  |  |  |  |  |  |  |
| C | *Rubus ursinus* | trailing blackberry | 66.7 | 0.6 | 0.5 |  | 0.1 |  | 0.1 | 2.0 | 1.0 |  | 2.0 |  |  |
| C | *Streptopus amplexifolius* | clasping twistedstalk | 11.1 | 0.1 |  | 0.1 |  |  |  |  |  |  |  |  |  |
| C | *Trientalis borealis* | broad-leaved starflower | 11.1 | 0.1 |  |  |  |  |  |  |  |  |  |  | 0.5 |
| C | *Trientalis borealis ssp. latifolia* | broad-leaved starflower | 77.8 | 0.1 |  | 0.1 | 0.1 |  | 0.1 | 1.0 | 0.1 |  | 0.1 | 0.1 |  |
| D | *Acer macrophyllum* | bigleaf maple | 11.1 | 0.1 |  |  |  |  |  |  |  |  |  |  | 0.1 |
| D | *Eurhynchium oreganum* | Oregon beaked-moss | 100.0 | 21.3 | 23.0 | 80.0 | 40.0 |  | 5.0 | 20.0 | 6.0 |  | 3.0 | 10.0 | 5.0 |
| D | *Eurhynchium praelongum* | slender beaked-moss | 22.2 | 0.2 |  | 1.0 | 1.0 |  |  |  |  |  |  |  |  |
| D | *Hylocomium splendens* | step moss | 88.9 | 28.9 | 2.0 | 10.0 | 55.0 |  |  | 5.0 | 3.0 |  | 80.0 | 80.0 | 25.0 |
| D | *Plagiothecium undulatum* | flat-moss | 22.2 | 0.3 |  |  |  |  |  |  |  |  |  | 2.0 | 1.0 |
| D | *Rhytidiadelphus loreus* | lanky moss | 44.4 | 1.4 |  |  |  |  |  | 10.0 | 1.0 |  | 2.0 |  | 0.1 |
| D | *Rhytidiadelphus triquetrus* | electrified cat's-tail moss | 22.2 | 0.9 |  |  | 8.0 |  |  | 0.1 |  |  |  |  |  |
| D | *Rhytidiopsis robusta* | pipecleaner moss | 11.1 | 0.3 |  | 3.0 |  |  |  |  |  |  |  |  |  |
| D | *Thuja plicata* | western redcedar | 11.1 | 0.1 |  |  |  |  |  |  |  |  |  |  | 0.1 |

Table S5: Concentration ([DNA]) and λ absorption ratios (260/280 and 260/230) of DNA extracted from forest floor and mineral soil samples. Each value represents the mean of 4 sampled plots (±SE).

|  |  | Forest floor | | |  | Mineral soil | | |
| --- | --- | --- | --- | --- | --- | --- | --- | --- |
| Transect | Zone | [DNA] (ng/ul) | 260/280 | 260/230 |  | [DNA] (ng/ul) | 260/280 | 260/230 |
| South | DF | 16.93 ±0.77 | 2.03 ±0.15 | 1.42 ±0.17 |  | 25.73 ±8.36 | 1.81 ±0.06 | 1.54 ±0.26 |
|  | TR | 18.12 ±3.26 | 2.01 ±0.19 | 1.51 ±0.23 |  | 24.61 ±4.33 | 1.83 ±0.11 | 1.65 ±0.23 |
|  | WH | 14.88 ±1.14 | 2.06 ±0.19 | 1.46 ±0.22 |  | 31.48 ±9.7 | 1.81 ±0.06 | 1.43 ±0.09 |
| Central | DF | 12.44 ±2.72 | 1.92 ±0.22 | 1.31 ±0.31 |  | 18.14 ±2.66 | 1.77 ±0.06 | 1.46 ±0.23 |
|  | TR | 14.78 ±1.46 | 2.05 ±0.15 | 1.62 ±0.09 |  | 22.63 ±5.4 | 1.89 ±0.03 | 1.09 ±0.04 |
|  | WH | 16.86 ±2.03 | 1.80 ±0.07 | 1.07 ±0.2 |  | 30.46 ±6.23 | 1.75 ±0.07 | 1.14 ±0.29 |
| North | DF | 14.28 ±2.9 | 2.13 ±0.16 | 1.13 ±0.26 |  | 17.51 ±5.62 | 1.84 ±0.07 | 1.01 ±0.18 |
|  | TR | 12.09 ±2.33 | 1.86 ±0.18 | 1.06 ±0.16 |  | 23.32 ±12.01 | 1.91 ±0.11 | 0.90 ±0.28 |
|  | WH | 15.26 ±1.66 | 1.87 ±0.12 | 0.98 ±0.11 |  | 17.88 ±0.69 | 1.80 ±0.09 | 1.25 ±0.13 |

Table S6: The significant^a^ probability-values^b^ of models and constraints in explaining vegetation cover, forest floor characteristics or mineral soil characteristics. Constraints are labelled with their respective *P*-values, or ‘n.s.’ in cases where these are not significant. Percent inertia explained by each model is included when models are significant. Mean annual soil temperature variables (TS, TS_max_, TS_min_) were included in analyses but not selected as significant constraints in any of our models.

|  |  | Responses | | | | |
| --- | --- | --- | --- | --- | --- | --- |
| Model | Constraints | Vegetation | Forest floor characteristics | | Mineral soil characteristics | |
| Constrained by site | Latitude | 0.03 | <0.001 | <0.001 | |  |
|  | Zone | n.s. | <0.001 | <0.001 | |  |
|  | Lat:Zone | n.s. | <0.001 | <0.001 | |  |
|  | % interia explained | 34.7 | 56.7 | 73 | |  |
|  | *P*-value of model | 0.033 | <0.001 | <0.001 | |  |
| Constrained by climate | MS | n.s. | 0.025 | 0.013 | |  |
|  | MS_min_ | n.s. | 0.002 | n.s. | |  |
|  | MS_max_ | 0.004 | n.s. | <0.001 | |  |
|  | PET | 0.035 | 0.003 | n.s. | |  |
|  | DD | n.s. | 0.001 | n.s. | |  |
|  | TA | n.s. | n.s. | 0.003 | |  |
|  | TA_min_ | n.s. | <0.001 | 0.001 | |  |
|  | TA_max_ | n.s. | <0.001 | 0.023 | |  |
|  | % interia explained | 34.7 | 56.7 | 73 | |  |
|  | *P*-value of model | 0.031 | <0.001 | <0.001 | |  |

^a^ α-level of 0.05

^b^ Analysed by *X^2^* comparisons of full and reduced models using permutation pseudo-F tests (9999 permutations) for RDA (for community structure) and CCA (for community composition); all responses were scaled to unit variance

Table S7: The significant^a^ probability-values^b^ of models and constraints explaining the structure and composition of microbial communities in forest floors. The microbial functional groups used as responses are designated by *18S*, *NifH* and *AmoA* for target fungal, nitrogen-fixing and ammonia-oxidizing bacteria communities, respectively, or ‘ALL’ when all three groups are used. Constraints selected by backwards elimination based on AIC-like statistics are labelled with their respective *P*-values, or ‘n.s.’ in cases where these are non-significant. Constraints not used in the models are marked by a dash. Percent inertia explained by each model is included when models are significant. Mean annual soil temperature variables (TS, TS_max_, TS_min_) were included in analyses but not selected as significant constraints in any of our models. Conditional models not analyzed due to a non-significant climatic or edaphic/vegetation model are labelled Not Applicable (N/A).

| Forest floor samples | | Responses | | | | | | | |
| --- | --- | --- | --- | --- | --- | --- | --- | --- | --- |
|  |  | Community Structure | | | | Community Composition | | | |
| Model | Constraints | ALL | *18S* | *NifH* | *AmoA* | ALL | *18S* | *NifH* | *AmoA* |
| a) Constrained by site | Latitude | n.s. | n.s. | n.s. | n.s. | <0.001 | <0.001 | 0.021 | n.s. |
|  | Zone | n.s. | n.s. | n.s. | n.s. | n.s. | n.s. | n.s. | n.s. |
|  | Lat:Zone | n.s. | n.s. | n.s. | 0.015 | 0.048 | n.s. | n.s. | n.s. |
|  | % interia explained | - | - | - | 36.8 | 30.7 | 33.2 | 30.9 | - |
|  | *P*-value of model | 0.083 | 0.088 | 0.68 | 0.016 | <0.001 | <0.001 | 0.035 | 0.53 |
| b) Constrained by climate | MS | - | - | - | - | 0.005 | 0.003 | n.s. | - |
|  | MS_min_ | n.s. | - | - | - | 0.007 | 0.033 | n.s. | - |
|  | MS_max_ | - | - | - | - | - | - | - | - |
|  | PET | - | n.s. | - | 0.014 | - | - | n.s. | - |
|  | DD | - | 0.03 | - | n.s. | 0.001 | - | n.s. | n.s. |
|  | TA | 0.004 | n.s. | n.s. | n.s. | - | 0.038 | n.s. | - |
|  | TA_min_ | 0.027 | - | - | n.s. | 0.024 | 0.007 | n.s. | n.s. |
|  | TA_max_ | - | 0.027 | - | 0.013 | 0.019 | n.s. | 0.005 | 0.021 |
|  | % interia explained | 18.4 | 27.1 | - | 30.2 | 22.3 | 23.2 | 29.2 | 14.8 |
|  | *P*-value of model | 0.001 | 0.019 | 0.69 | 0.005 | <0.001 | <0.001 | 0.013 | 0.015 |
| c) Constrained by edaphic characteristics and vegetation cover | Fine | - | - | - | n.s. | - | - | 0.004 | - |
|  | %C | - | - | 0.02 | - | - | - | - | - |
|  | %N | 0.026 | 0.013 | - | - | - | - | - | - |
|  | C/N | - | - | - | - | - | n.s. | - | 0.022 |
|  | pH | n.s. | 0.023 | - | n.s. | 0.006 | - | - | - |
|  | Coarse | - | n.s. | - | - | - | - | - | - |
|  | Co/F | - | - | - | - | - | - | - | - |
|  | Salal | - | - | - | - | - | - | - | 0.013 |
|  | Ferns | - | n.s. | - | - | - | - | - | - |
|  | Oregon grape | - | - | - | - | - | - | - | - |
|  | Vanilla leaf | - | - | - | - | - | - | - | - |
|  | Moss | - | - | - | - | - | - | <0.001 | - |
|  | % interia explained | 12.2 | 34.5 | 14 | - | 4.9 | - | 17 | 13.2 |
|  | *P*-value of model | 0.012 | 0.014 | 0.02 | >0.05 | 0.006 | 0.055 | <0.001 | 0.002 |
| d) Constrained by climate, conditioned by edaphic characteristics and vegetation cover | MS | - | - | N/A | N/A | n.s. | N/A | n.s. | - |
|  | MS_min_ | n.s. | - | N/A | N/A | 0.007 | N/A | n.s. | - |
|  | MS_max_ | - | - | N/A | N/A | - | N/A | - | - |
|  | PET | - | n.s. | N/A | N/A | - | N/A | n.s. | - |
|  | DD | - | n.s. | N/A | N/A | 0.002 | N/A | n.s. | n.s. |
|  | TA | 0.027 | n.s. | N/A | N/A | - | N/A | n.s. | - |
|  | TA_min_ | n.s. | - | N/A | N/A | 0.04 | N/A | n.s. | n.s. |
|  | TA_max_ | - | n.s. | N/A | N/A | 0.014 | N/A | n.s. | 0.023 |
|  | % interia explained by constraints | - | - | N/A | N/A | 20.4 | N/A | - | - |
|  | % inertia explained by conditions | - | - | N/A | N/A | 4.9 | N/A | - | - |
|  | *P*-value of model | 0.056 | 0.64 | N/A | N/A | <0.001 | N/A | 0.33 | 0.076 |

^a^ α-level of 0.05

^b^ Analysed by *X^2^* comparisons of full and reduced models using permutation pseudo-F tests (9999 permutations) for RDA (for community structure) and CCA (for community composition); all responses were scaled to unit variance

Table S8: The significant^a^ probability-values^b^ of models and constraints explaining the structure and composition of microbial communities in mineral soils. The microbial functional groups used as responses are designated by *18S*, *NifH* and *AmoA* for target fungal, nitrogen-fixing and ammonia-oxidizing bacteria communities, respectively, or ‘ALL’ when all three groups are used. Constraints selected by backwards elimination based on AIC are labelled with their respective *P*-values, or ‘n.s.’ in cases where these are non-significant. Constraints not used in the models are marked by a dash. Percent inertia explained by each model are included when models are significant. Mean annual soil temperature variables (TS, TSmax, TSmin) were included in analyses but not selected as significant constraints in any of our models. Conditional models not analyzed due to a non-significant climatic or edaphic/vegetation model are labelled Not Applicable (N/A).

| Mineral soil |  | Responses | | | | | | | |
| --- | --- | --- | --- | --- | --- | --- | --- | --- | --- |
|  |  | Community Structure | | | | Community Composition | | | |
| Model | Constraints | ALL | *18S* | *NifH* | *AmoA* | ALL | *18S* | *NifH* | *AmoA* |
| a) Constrained by site | Latitude | <0.001 | 0.012 | <0.001 | n.s. | <0.001 | <0.001 | <0.001 | 0.029 |
|  | Zone | 0.004 | n.s. | 0.041 | n.s. | <0.001 | 0.016 | <0.001 | n.s. |
|  | Lat:Zone | 0.01 | 0.046 | n.s. | n.s. | 0.001 | 0.018 | 0.001 | n.s. |
|  | % interia explained | 51.5 | 45.3 | 64.5 | - | 33.1 | 31 | 44.6 | 29.9 |
|  | *P*-value of model | <0.001 | 0.008 | <0.001 | 0.052 | <0.001 | <0.001 | <0.001 | 0.015 |
| b) Constrained by climate | MS | 0.01 | - | 0.026 | 0.004 | <0.001 | 0.007 | <0.001 | 0.014 |
|  | MS_min_ | - | - | - | n.s. | n.s. | - | - | - |
|  | MS_max_ | <0.001 | 0.001 | 0.048 | - | - | - | 0.006 | - |
|  | PET | - | - | - | - | n.s. | - | - | - |
|  | DD | n.s. | - | - | 0.025 | 0.008 | - | 0.036 | - |
|  | TA | - | - | - | - | 0.024 | - | 0.016 | - |
|  | TA_min_ | - | - | - | - | 0.002 | 0.024 | - | - |
|  | TA_max_ | n.s. | n.s. | - | - | <0.001 | 0.038 | n.s. | - |
|  | % interia explained | 25.9 | 41.7 | 21.9 | 32.5 | 29.6 | 12.6 | 29.2 | 6.5 |
|  | *P*-value of model | <0.001 | 0.004 | 0.011 | <0.001 | <0.001 | <0.001 | <0.001 | 0.012 |
| c) Constrained by edaphic characteristics and vegetation cover | Fine | 0.03 | 0.006 | - | n.s. | 0.003 | <0.001 | <0.001 | - |
|  | %C | - | - | - | - | 0.001 | - | 0.009 | - |
|  | %N | - | 0.047 | - | - | 0.008 | - | - | - |
|  | C/N | 0.002 | 0.024 | 0.005 | - | - | 0.022 | - | - |
|  | pH | <0.001 | - | <0.001 | 0.003 | <0.001 | - | <0.001 | 0.007 |
|  | Coarse | - | - | - | - | - | - | - | - |
|  | Co/F | - | n.s. | - | - | - | - | - | - |
|  | Salal | - | - | - | - | - | - | 0.017 | - |
|  | Ferns | 0.011 | - | - | 0.012 | - | - | - | - |
|  | Oregon grape | - | - | - | - | - | - | - | - |
|  | Vanilla leaf | - | - | - | - | - | - | - | - |
|  | Moss | - | - | - | - | - | - | - | - |
|  | % interia explained | 34.7 | 34.5 | 38.4 | 30.7 | 19.4 | 9.4 | 25 | 6.5 |
|  | *P*-value of model | <0.001 | 0.001 | 0.001 | 0.002 | <0.001 | <0.001 | <0.001 | 0.007 |
| d) Constrained by climate, conditioned by edaphic characteristics and vegetation cover | MS | n.s. | - | n.s. | n.s. | <0.001 | 0.006 | 0.001 | n.s. |
|  | MS_min_ | - | - | - | n.s. | n.s. | - | - | - |
|  | MS_max_ | n.s. | n.s. | n.s. | - | - | - | 0.003 | - |
|  | PET | - | - | - | - | n.s. | - | - | - |
|  | DD | n.s. | - | - | 0.039 | 0.04 | - | n.s. | - |
|  | TA | - | - | - | - | 0.01 | - | 0.001 | - |
|  | TA_min_ | - | - | - | - | n.s. | 0.023 | - | - |
|  | TA_max_ | n.s. | n.s. | - | - | 0.019 | n.s. | n.s. | - |
|  | % interia explained by constraints | - | - | - | - | 23 | 11.5 | 21.4 | - |
|  | % inertia explained by conditions | - | - | - | - | 19.4 | 9.4 | 25 | - |
|  | *P*-value of model | 0.198 | 0.29 | 0.135 | 0.109 | <0.001 | 0.001 | <0.001 | 0.067 |

^a^ α-level of 0.05

^b^ Analysed by *X^2^* comparisons of full and reduced models using permutation pseudo-F tests (9999 permutations) for RDA (for community structure) and CCA (for community composition); all responses were scaled to unit variance

Table S9: Number of Operational Taxonomic Units (OTU) and Shannon's diversity indices (H') of microbial communities from DNA extracted from forest floor and mineral soil samples. Functional groups of fungi, nitrogen-fixing (N fix) bacteria and ammonia-oxidizing (AO) bacteria were amplified using 18S, Nif-H-universal and amoA primer sets, respectively. Each value represents the mean of 4 sampled plots (±SE).

|  |  | Forest floor | | | | | | | |
| --- | --- | --- | --- | --- | --- | --- | --- | --- | --- |
|  |  | Fungi | | N fix Bacteria | | AO Bacteria | | | |
| Transect | Zone | OTU | H' | OTU | H' | OTU | | H' | |
| South | DF | 12.75 ±1.25 | 1.10 ±0.06 | 2.75 ±0.63 | 0.50 ±0.29 | 0.00 | | 0.30 ±0.11 | |
|  | TR | 13.00 ±0.91 | 1.11 ±0.12 | 1.25 ±0.63 | 1.00 ±1 | 0.14 ±0.14 | | 0.11 ±0.11 | |
|  | WH | 14.25 ±0.63 | 1.26 ±0.1 | 2.00 ±0.71 | 2.75 ±1.6 | 0.28 ±0.17 | | 0.19 ±0.12 | |
| Central | DF | 9.50 ±2.63 | 0.75 ±0.25 | 2.00 ±1.35 | 7.00 ±2.35 | 0.65 ±0.16 | | 0.19 ±0.19 | |
|  | TR | 13.00 ±0.91 | 1.14 ±0.07 | 0.25 ±0.25 | 0.00 | 0.00 | | 0.00 | |
|  | WH | 14.25 ±0.85 | 1.33 ±0.19 | 1.25 ±0.63 | 1.25 ±1.25 | 0.16 ±0.16 | | 0.11 ±0.11 | |
| North | DF | 12.25 ±0.48 | 0.98 ±0.09 | 2.25 ±1.03 | 1.50 ±0.29 | 0.12 ±0.07 | | 0.25 ±0.15 | |
|  | TR | 13.25 ±1.31 | 0.90 ±0.13 | 0.75 ±0.48 | 0.00 | 0.00 | | 0.04 ±0.04 | |
|  | WH | 11.25 ±1.11 | 0.83 ±0.12 | 0.50 ±0.5 | 0.00 | 0.00 | | 0.06 ±0.06 | |
|  |  | Mineral soil | | | | | | |  |
|  |  | Fungi | | N fix Bacteria | | AO Bacteria | | |  |
| Transect | Zone | OTU | H' | OTU | H' | OTU | H' | |  |
| South | DF | 14.50 ±0.29 | 1.28 ±0.12 | 9.75 ±0.85 | 0.89 ±0.04 | 1.00 ±0.41 | 0.07 ±0.07 | |  |
|  | TR | 11.50 ±0.29 | 0.98 ±0.09 | 6.00 ±1.68 | 0.59 ±0.14 | 1.25 ±1.25 | 0.13 ±0.13 | |  |
|  | WH | 12.25 ±0.75 | 1.09 ±0.08 | 9.25 ±1.65 | 0.74 ±0.06 | 1.75 ±0.63 | 0.23 ±0.1 | |  |
| Central | DF | 11.25 ±1.03 | 0.89 ±0.16 | 9.75 ±1.11 | 0.88 ±0.05 | 7.25 ±2.06 | 0.70 ±0.16 | |  |
|  | TR | 12.00 ±0.91 | 1.07 ±0.03 | 7.25 ±0.48 | 0.69 ±0.02 | 3.25 ±2.36 | 0.33 ±0.22 | |  |
|  | WH | 12.50 ±1.19 | 1.12 ±0.11 | 6.50 ±3.01 | 0.52 ±0.2 | 0.75 ±0.48 | 0.07 ±0.07 | |  |
| North | DF | 10.25 ±0.85 | 0.84 ±0.02 | 2.25 ±0.75 | 0.22 ±0.13 | 0.50 ±0.5 | 0.07 ±0.07 | |  |
|  | TR | 10.00 ±0.71 | 0.76 ±0.04 | 2.00 ±0.91 | 0.21 ±0.12 | 1.25 ±0.48 | 0.13 ±0.08 | |  |
|  | WH | 13.00 ±0.91 | 1.00 ±0.04 | 0.50 ±0.5 | 0.02 ±0.02 | 0.75 ±0.48 | 0.04 ±0.04 | |  |

**1.2 Supplementary Figures**

Figure S1: Technical replicates of the PCR-DGGE process for amplification and separation of fungal-specific partial fragments of 18S rDNA (A), *Nif-H* genes (B) and *AmoA* genes (C). Each column represents a unique PCR reaction performed on the same DNA sample extracted from the field, while each pair of columns represent PCR amplicons separated on the same DGGE gel.

Figure S2: RDA of tree **(A)** and moss **(B)** cover at sites, constrained by latitude and zone. Polygons represent k-means clustering using RDA components 1 and 2. Tree species include *Thuja plicata* (Tp), *Tsuga heterophylla* (Th) and *Pseudotsuga menziesii var. menziesii* (Pm)*.* Moss species include *Eurhynchium oreganum* (Eo), *Eurhynchium praelongum* (Ep), *Hylocomium splendens* (Hs), *Plagiothecium undulatum* (Pu), *Rhytidiadelphus loreus* (Rl), *Rhytidiadelphus triquetrus* (Rt) *and Rhytidiopsis robusta* (Rr). All responses were scaled to unit variance. Percent inertia explained by each RDA component is in parenthesis next to axis label.

Figure S3: PCNMs of spatial coordinates of sampled plots selected in microbial community response models. Increasing PCNM numbers represent smaller spatial scales (Borcard et al. 2004). The 4 plot points at each site are not distinguishable at this scale. PCNM 1 and 3 correlated with latitude (*r* = 0.8673 and 0.4684 respectively, *P* < 0.01) while PCNM 4 and 5 correlated with zone (*r* = -0.6568 and -0.5362 respectively, *P* < 0.01). PCNM 2 did not correlate with any measure environmental variables (*P* > 0.05).

**References**

Borcard, D., Legendre, P., Avois-Jacquet, C., Tuomisto, H., 2004. Dissecting the spatial structure of ecological data at multiple scales. Ecology 85, 1826-1832.

Meidinger, D. V. and J. Pojar. 1991. Ecosystems of British Columbia. BC Ministry of Forests, Victoria, BC.
